# Deguelin’s Anticancer Bioactivity: Challenges and Opportunities in Medicinal Chemistry

**DOI:** 10.3389/fphar.2025.1571452

**Published:** 2025-06-26

**Authors:** Wenjie Jiang, Jing Zeng, Li Guo, Jianyou Shi, Yang Lei

**Affiliations:** ^1^ Department of Pharmacy, Personalized Drug Therapy Key Laboratory of Sichuan Province, Sichuan Academy of Medical Science and Sichuan Provincial People’s Hospital, School of Medicine, University of Electronic Science and Technology of China, Chengdu, China; ^2^ School of Food and Bioengineering, Xihua University, Chengdu, Sichuan, China; ^3^ The State Key Laboratory of Southwestern Chinese Medicine Resources, Department of Pharmacy, Chengdu University of Traditional Chinese Medicine, Chengdu, Sichuan, China

**Keywords:** deguelin, deguelin derivatives, cancer, heat-shock proteins, structure-activity relationship, HSP90 inhibitor

## Abstract

Deguelin is a natural isoflavone derived from the rotenone family, commonly found in plants belonging to the Derris and Grifola genera. Comprehensive research has underscored its considerable promise in oncological treatment. Deguelin demonstrates anticancer activity by suppressing cell proliferation and promoting apoptosis *via* the modulation of critical signaling pathways, such as NF-κB, Wnt, and AMPK pathways. Moreover, deguelin demonstrates several biological activities, including cell cycle arrest, autophagy modulation, anti-angiogenic and anti-metastatic capabilities, along with antioxidant and anti-inflammatory actions. Notwithstanding these encouraging benefits, the practical utilization of deguelin has been impeded by its volatility, possible neurotoxicity, and other detrimental consequences. This review initially examines the antitumor biological actions of deguelin, incorporating recent discoveries regarding its methods of action. It subsequently consolidates studies on structural alterations intended to enhance the efficacy of deguelin while mitigating its toxicity, and offers a summary of the structure-activity connections of its derivatives. The review seeks to further research on deguelin and guide the development of more efficacious derivatives for prospective clinical applications. This study seeks to establish a robust basis for the advancement of deguelin as a potential chemopreventive agent for cancer.

## Graphical Abstract

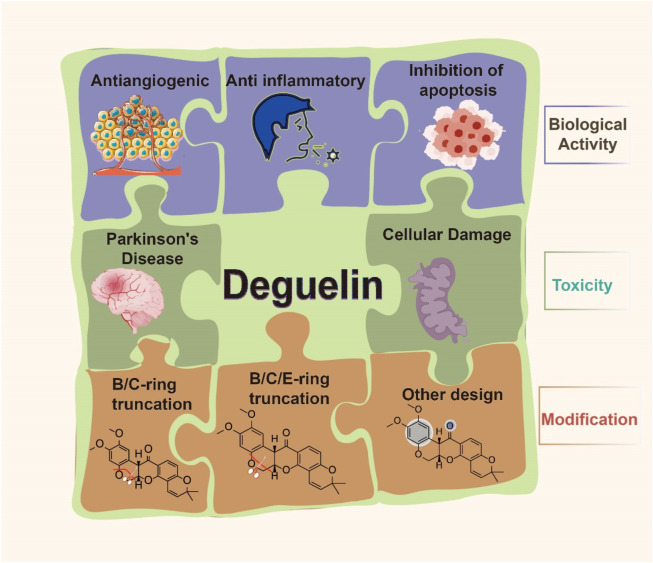

## Highlights

- Describe the function, and role of deguelin in cancer and other bioactivities.
- Summarize the current (2014 - present) progress and characteristics of deguelin derivatives.
- Classify optimization strategies and structure - activity relationships of different HSP90 inhibitors.
- Offer suggestions for the further medicinal development of deguelin analogs.

## 1 Introduction

Deguelin, a naturally occurring rotenoid, is derived from a wide range of plant species. The chemical definition is (7aS,13aS)-13,13a-dihydro-9,10-dimethoxy-3,3-dimethyl-3H-bis [1] benzopyrano [3,4-b:6ʹ,5ʹ-3] pyran-7 (7aH)-one. It has been identified by sources such as the bark of Mundulea sericea, the roots of *Derris trifoliata* Lour., the young leaves of Tephrosia vogelii Hook. F., and the seeds of Millettia pachycarpa Benth. Despite its biological significance, the extraction efficiency of natural deguelin from these plants is still insufficient for large-scale applications. Chemical synthesis is a viable and efficient option. The introduction of the initial synthetic method for rotenone in the 1960s led to the lack of convenient access to natural bioactive compounds that hindered further exploration into the synthesis of rotenoids (Miyano, 1965; Miyano et al., 1961). The synthesis of (−)-deguelin from rotenone as a precursor has been documented. In 2010, Garcia J and his associates first accomplished the asymmetric synthesis of (−)-deguelin (Garcia et al., 2010).

**FIGURE 1:**
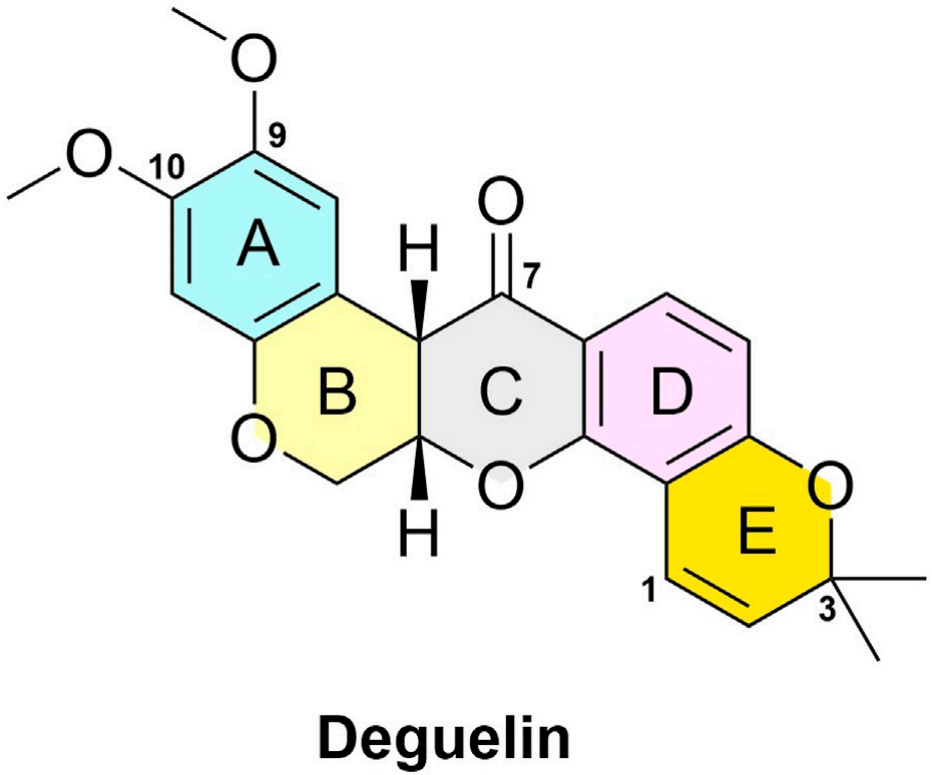
The structure of the deguelin.

As depicted in Figure 1, the structure of deguelin is characterized by a rigid pentacyclic framework comprising five rings. Dimethoxy-substituted dihydrobenzopyran moiety is formed by the AB rings. The C - ring, which is centrally positioned, serves as a crucial bridge within the structure. The DE rings form a dimethylbenzopyran structure, with the E-ring showing a chromene configuration. The BC rings, characterized as six-membered heterocycles, have garnered considerable attention for structural modification owing to their potential for functional optimization. The DE-ring framework functions as a distinctive pharmacophore and is essential for preserving the compound’s overall bioactivity. The presence of methoxy groups on the A-ring is crucial for activity. Furthermore, the bridging chain connecting the B and C rings is essential, although there exists a degree of flexibility in its assembly. Importantly, modifications at the C7 position are notably significant, as substituents in this area greatly affect the compound’s antitumor efficacy against cancer cells.

In the coming decades, there is a significant increase in the number of cancer cases, which is a leading cause of death worldwide (Siegel et al., 2022). Over the past few decades, an increasing body of evidence has demonstrated that natural products, as traditional sources for modern drug discovery and potential drug leads, play an indispensable role in cancer treatment due to their unique structural, chemical, and biological diversity (Atanasov et al., 2021; Newman and Cragg, 2020; Porras et al., 2021). Diverse anticancer properties can be exhibited by a variety of naturally occurring bioactive substances (Salehi et al., 2020). Vinblastine, vincristine, etoposide, paclitaxel, and camptothecin were all originally derived from the plant kingdom (Cragg and Pezzuto, 2016).

Comprehensive studies have shown the significant anticancer properties of deguelin. Both *in vitro* and *in vivo* studies have highlighted its exceptional pharmacological properties in combating various malignancies. Deguelin exerts its antitumor effects primarily through the induction of apoptosis, the arrest of the cell cycle, and the inhibition of angiogenesis in various cancers, including gastric, lung, and breast carcinomas (Lee et al., 2010). Research indicates that it facilitates apoptosis in gallbladder carcinoma cells (SGC - 996), human colorectal carcinoma cells (RKO), and B- chronic lymphocytic leukemia cells (Chen et al., 2019). Furthermore, it suppresses the proliferation of HTLV-1-transformed KUT-1 cells (Ito et al., 2010). The anticancer properties of deguelin can be ascribed to its engagement with various molecular targets, which effectively inhibit tumor growth *via* multiple signaling pathways. The pathways encompass phosphatidylinositol 3-kinase (PI3K), serine/threonine protein kinase B (Akt), mammalian target of rapamycin (mTOR), mitogen-activated protein kinase (MAPK), nuclear factor-κB (NF-κB), extracellular signal-regulated kinase (ERK), and matrix metalloproteinases (MMPs). Furthermore, apoptosis-executing proteins, including Caspase-3, Caspase-8, and Caspase-9, play a crucial role in the tumor inhibition mediated by deguelin. Additionally, deguelin specifically targets hypoxia-inducible factor-1α (HIF-1α), focal adhesion kinase (FAK), vascular endothelial growth factor (VEGF), VEGF receptor (VEGFR), platelet endothelial cell adhesion molecule-1 (PECAM-1), and NIMA-related kinase 2 (Nek2). This targeting plays a significant role in its anti-angiogenic properties and its ability to inhibit tumor metastasis. In addition to its anticancer properties, deguelin demonstrates significant anti-inflammatory activity. Inhibition of heat shock protein 90 (Hsp90) effectively mitigates chronic inflammation, encompassing the inflammatory responses associated with allergic asthma. Initial preclinical investigations have suggested that deguelin may serve as a promising anticancer agent, functioning effectively both as a standalone treatment and as a synergistic enhancer of chemotherapy and radiotherapy. The exploration of the molecular targets of deguelin could provide significant insights for the advancement of innovative anticancer therapies. Deguelin’s ability to induce apoptosis while inhibiting malignant transformation and the proliferation of tumor cells establishes it as a carotenoid with significant potential for chemotherapeutic and chemopreventive applications. The findings underscore the necessity of accelerating drug development initiatives for deguelin as a formidable anticancer agent.

Prior research on the various biological activities of deguelin has highlighted its potential toxic side effects, particularly at elevated concentrations. Deguelin has been shown to inhibit the activity of tyrosine hydroxylase in dopaminergic neurons, resulting in Parkinsonian-like syndromes. It is recognized as an inhibitor of ubiquinone oxidoreductase within mitochondrial complex I (Okun et al., 1999), leading to impaired mitochondrial function and subsequent cellular damage. Caboni et al. (2004) suggested that the Parkinsonian effects of deguelin primarily arise from the inhibition of mitochondrial complex I within the respiratory chain. In a similar vein, Kim et al. (2008) indicated that deguelin not only induces symptoms akin to Parkinson’s disease (PD) but also influences the Src/signal transducers and activators of transcription (STAT) signaling pathway. The activation of Src, induced by deguelin, results in the phosphorylation of α-synuclein, which is a significant event in the pathogenesis of Parkinson’s disease. Deguelin, a specific inhibitor of Hsp90, exhibits significant cytotoxicity that also affects cellular and tissue integrity. The toxic effects present considerable challenges to the clinical application of deguelin, underscoring the need for structural optimization to mitigate toxicity while preserving its biological activity. Researchers have focused on improving the stability and selectivity of deguelin by implementing structural modifications to address this issue. This has resulted in the synthesis of various derivatives and the accumulation of comprehensive structure-activity relationship (SAR) data.

**FIGURE 2:**
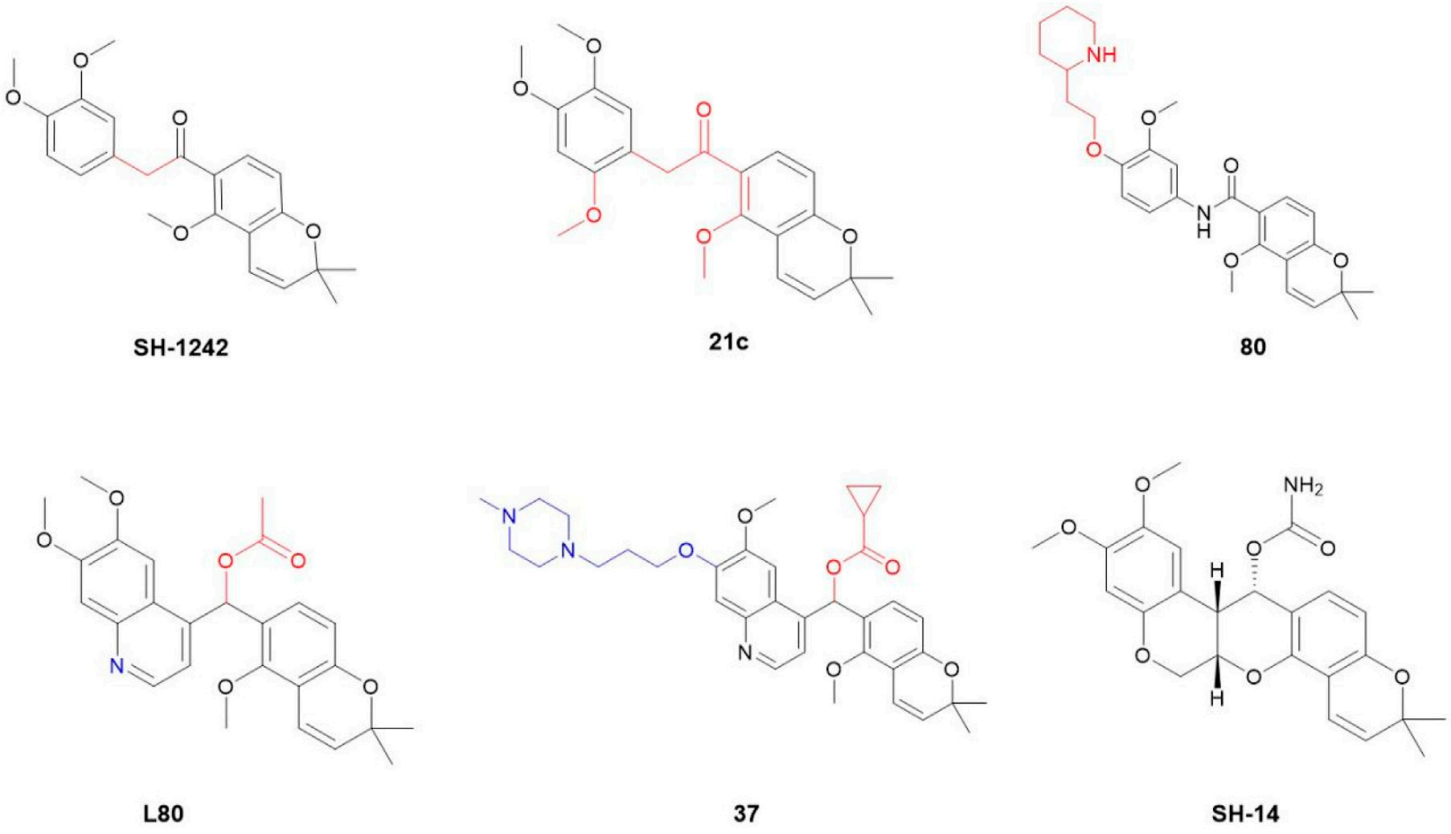
Chemical structures of some representative deguelin derivatives.

Given the rigid pentacyclic structure of deguelin, researchers have investigated multiple strategies for structural modification, including alterations to individual rings and the execution of localized oxidation and reduction processes (Figure 2). Deguelin’s principal anticancer target, Hsp90, possesses multiple binding sites. Conventional Hsp90 inhibitors typically focus on the N-terminal domain, which is linked to significant toxicity. Recent studies have shifted their attention to the C-terminal domain of Hsp90, as compounds that interact with this region demonstrate reduced toxic effects. Su - Chan Lee and colleagues synthesized the deguelin derivative L80 (Lee et al., 2015), which demonstrated markedly lower cytotoxicity in HT - 22 cells compared to deguelin, while preserving its anticancer activity through interactions with critical residues in the Hsp90 C - terminal domain. Following this accomplishment, the same research group advanced the development of SH - 1242 (Lee et al., 2016), a derivative that maintains significant antitumor efficacy while substantially minimizing neurotoxic effects. The advancements demonstrate the capacity of structural optimization to overcome the constraints of deguelin, thereby facilitating its safer and more effective therapeutic use.

This review aims to provide a thorough summary of the recent advancements in the research surrounding deguelin and its derivatives. We will specifically highlight the latest advancements in clarifying the antitumor activities of deguelin. Concurrently, we will conduct a thorough examination of the structural modification strategies and the SAR of its derivatives. This review aims to serve as a valuable resource for researchers engaged in systematic and comprehensive investigations of deguelin and its derivatives. This approach is anticipated to promote the advancement of innovative and efficient candidate molecules, thereby expediting their transition into clinical applications.

## 2 Pharmacokinetic properties of deguelin

Udeani et al. investigated the pharmacokinetics of deguelin in rats. Anesthetized rats received a single dose of deguelin at 0.25 mg/kg (intravenous injection) or 4 mg/kg (intragastric administration) (Udeani et al., 2001), and blood samples were collected over 24 h, with monitoring continued for 5 days. The results demonstrated that deguelin follows a three-compartment model with first-order elimination kinetics. It exhibited a long mean residence time (6.98 h) and a terminal half-life of 9.26 h, supporting its potential for once-daily dosing. The large volume of distribution (3.42 L/kg) likely reflects extensive tissue distribution, particularly in the heart, liver, and kidneys. Excretion studies revealed that deguelin is primarily eliminated *via* feces (58.1%), with only 14.4% excreted in urine. Notably, approximately 1.7% and 0.4% of the administered dose was excreted unchanged in feces and urine, respectively. Toxicity data showed species-dependent variability in median lethal doses (LD50): 300 mg/kg in mice, 3,200 mg/kg in rabbits, and 980 mg/kg in rats. These differences underscore the importance of interspecies variation in toxicity assessment. Given the limited pharmacokinetic data available, further studies in additional animal models are recommended to comprehensively evaluate deguelin’s pharmacokinetic parameters, including its bioavailability.

## 3 Deguelin and cancer

**FIGURE 3:**
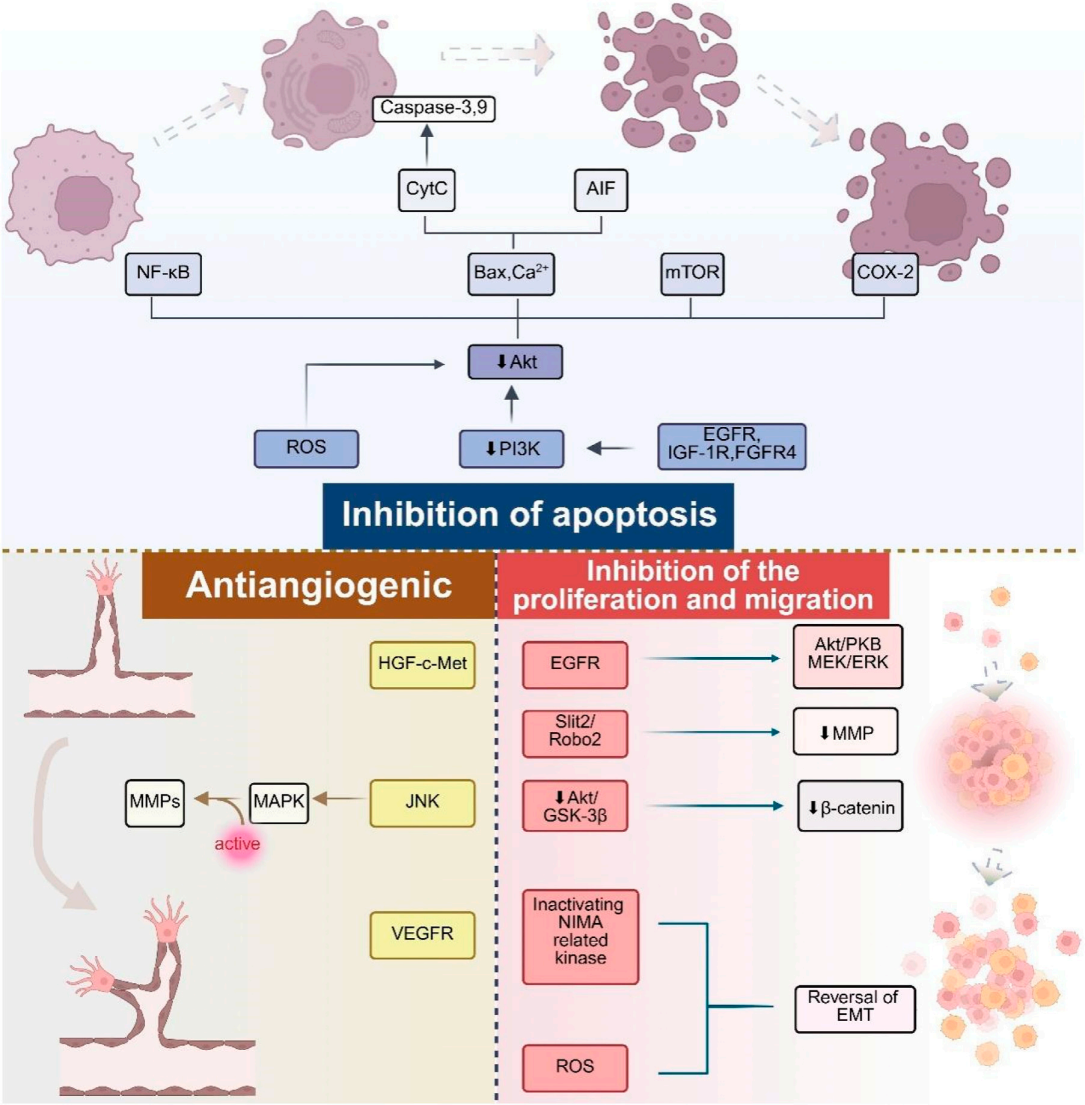
Major biological activities and mechanisms of deguelin.

Research has shown that deguelin can significantly inhibit the growth of various tumor cells (Tuli et al., 2021; Varughese et al., 2019). The primary mechanism through which it demonstrates anticancer activity involves the induction of apoptosis and cell cycle arrest, alongside anti-angiogenesis, antitumor metastasis, anti-inflammatory, and antioxidant effects. The mechanisms were thoroughly investigated owing to their involvement in various targeted pathological pathways (Figure 3).

### 3.1 Induction of apoptosis and cell cycle arrest

**FIGURE 4:**
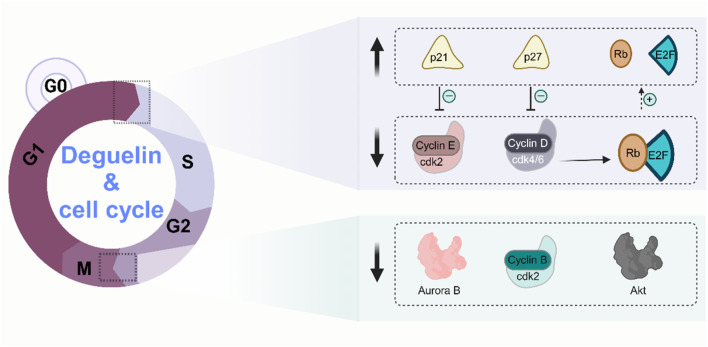
Molecular mechanisms of cell cycle arrest by deguelin.

Apoptosis is an essential mechanism for sustaining cellular homeostasis in multicellular organisms, characterised by controlled cell death (Aggarwal et al., 2019). Nonetheless, dysregulation of the apoptotic pathway is frequently linked to the progression of diverse tumors (Tuli et al., 2020). Multiple investigations have elucidated the involvement of Derrisquinone in cellular death *via* various pathways. In non-small cell lung cancer cells, rotenone demonstrated its ability to activate the mitochondrial-mediated apoptosis pathway by inhibiting the PI3K/Akt signalling pathway, upregulating the apoptosis regulator p53 upregulated modulator of apoptosis (PUMA), and augmenting the activity of Bcl-2-associated X (Bax) (Wang et al., 2018). Furthermore, Lu’s (Lv, 2020) research team demonstrated that deguelin and its derivative W-2 may halt the cell cycle of A549 and H1299 cells at the G2/M phase in non-small cell lung cancer. The cell scratch assay and Transwell migration assay demonstrated that deguelin and W-2 significantly and concentration-dependently inhibited the migration and invasion of these 2 cell types (p < 0.01). This inhibitory action is partially ascribed to their efficient inhibition of the PI3K/Akt/mTOR cell signalling pathway activation. The PI3K/Akt/mTOR signaling pathway, as one of the critical components in human tumor signaling networks, is extensively involved in regulating fundamental cellular processes such as proliferation, apoptosis, migration, and invasion (Beck et al., 2014). Numerous studies have demonstrated the hyperactivation of key protein molecules within the PI3K/Akt/mTOR pathway across various malignant tumors, including hepatocellular carcinoma, breast cancer, and glioblastoma (Stjernström et al., 2014; Wang et al., 2014). Yu et al. demonstrated the significant significance of deguelin in suppressing Aurora B kinase activity in esophageal squamous cell carcinoma cells. Aurora B kinase is essential for facilitating mitosis (Yu et al., 2017). The mechanism of cell cycle arrest by deguelin is shown in Figure 4.

In lung adenocarcinoma A549 cells, deguelin reduced the expression of MMP-2 and MMP-9 by downregulating slit guidance ligand 2/roundabout 2 receptor (slit2/robo2) and its downstream targets, which in turn affected the amount and morphology of cytoskeletal protein F-actin, and ultimately reduced the proliferation, invasion and migration ability of cells (Zhou H. H, et al., 2020). *In vitro* and *in vivo* studies on NCI-H520 and SK-MES-1 human lung squamous cell carcinoma cell lines showed that deguelin could downregulate the expression of galectin-1 in a time-concentration-dependent manner, thereby inhibiting the MAPK signaling pathway and inducing the apoptosis of lung squamous cell carcinoma cells (Yan et al., 2016). In breast cancer, deguelin effectively induced apoptosis of cancer cells by downregulating anti-apoptotic signaling pathways such as PI3K/Akt (Chu et al., 2011). Similarly, in ovarian cancer SKOV3 cells, deguelin inhibited cell proliferation and induced apoptosis by upregulating the expression of Kruppel like factor 15 (Klf15) (Zhao et al., 2022). In addition, the study of sun et al. also showed that when deguelin treated multiple myeloma cells *in vitro*, it could affect the mRNA expression regulating the cell cycle by regulating Akt and p38 MAPK signaling pathways, induce apoptosis and arrest the cells at the G2/M mitotic stage, thereby significantly reducing the growth, migration and invasion ability of these cells (Sun et al., 2024). Cai’s team focused on gallbladder cancer (Wang, 2020), and they used three different concentrations of deguelin to treat GBC-SD and SGC-996 gallbladder cancer cell lines, and found that deguelin could inhibit PTEN/PI3K/AKT signaling pathway and reverse epithelial mesenchymal transition (EMT) *in vitro*, and inhibit the proliferation, migration and invasion of gallbladder cancer cells in a dose-dependent manner. PTEN, a gene essential for maintaining normal cellular functions, is also one of the most frequently mutated tumor suppressor genes in human cancers. It plays a critical regulatory role in the growth and progression of various tumors (Li, Meng, et al., 2018, Wang et al., 2018). EMT represents an early adaptive change in tumor cells that acquire migratory and invasive properties for distant metastasis, primarily through the modulation of specific protein levels (Vaquero et al., 2017). The EMT process plays a pivotal role in promoting invasion and metastasis across multiple cancer types (Cao et al., 2019; Friedl and Wolf, 2003).

By establishing a nude mouse model of gallbladder cancer GBC-SD cell transplantation, they also confirmed that deguelin also has the ability to inhibit the tumorigenesis of gallbladder cancer cells *in vivo*. In addition, deguelin also exhibited significant anticancer activity in prostate cancer. In LNCaP and PC-3 human prostate cancer cells, deguelin promoted the protein degradation of β-catenin and reduced its accumulation in the nucleus by downregulating the phosphorylation levels of Akt and GSK-3 β, thereby inhibiting the transcriptional activation of β-catenin non responsive genes, thereby effectively inhibiting the growth, proliferation, invasion and migration of prostate cancer cells (Thamilselvan et al., 2011). Meanwhile, deguelin can also inhibit the expression of proliferation related proteins (such as cyclin D1 and c-myc) and anti-apoptotic proteins (such as Mcl-1, Bcl-xL and Survivin) in prostate cancer cells (Fang et al., 2024). *In vitro* and *in vivo* studies also showed that deguelin has significant anticancer activity against various tumor types such as gastric cancer MGC-803 and MKN-45 cells (Kang et al., 2018; Lin et al., 2021), and can induce programmed necrosis in gastric cancer SGC7901/VCR cells (Lz et al., 2018).

In extensive cancer research, deguelin mediated apoptotic events have also been shown to rely on NF-κB inhibitory κB (IκB) kinase (IKK) anti-apoptotic signaling pathway (Chen et al., 2006; Dat et al., 2008; Li et al., 2009). NF-κB is involved in inflammation mediated cancer progression, and its basic function is to upregulate the expression of anti-apoptotic proteins to inhibit cell death (Chen et al., 2006). Several studies have shown that deguelin treatment can inhibit NF-κB activation, thereby inducing apoptosis in multiple cell types. For example, Qian et al. (2024) showed that deguelin significantly reduced the proliferation activity of glioblastoma multiforme (GBM) cells and effectively inhibited the migration of GBM cells by promoting cell cycle arrest at G2/M stage and inducing apoptosis. Further RNA SEQ analysis revealed that the expression of chemokine CCL2 (its coding gene is CCL2) was significantly downregulated in deguelin treated GBM cells, and CCL2 is known to be closely associated with NF-κB signaling pathway. In addition, deguelin not only inhibited the CCL2/NF-κB signaling pathway, but also exogenous supplementation of CCL2 could partially reverse the growth inhibitory effect of deguelin on GBM cells by upregulating NF-κB activity. Particularly important, in the experiment of the allogeneic intracranial orthotopic GBM model, deguelin showed the ability to inhibit tumor growth and inhibit GBM angiogenesis by inhibiting the CCL2/NF-κB pathway *in vivo*. These findings strongly suggest that deguelin exerts a significant anti GBM effect by targeting and inhibiting the CCL2/NF-κB pathway. In addition, deguelin also exhibited extensive inhibitory effects on the expression of key downstream targets of NF-κB, including anti-apoptotic protein family members such as Bcl-2, Bcl-xL, Bcl-2-related protein A1 (Bfl-1), as well as apoptosis protein inhibitor 1/2 (IAP1/2) and tumor necrosis factor (TNF) receptor related factor 1 (TRAF-1) (Dat et al., 2008). In addition to the above pathways, deguelin can also specifically target the amp activated protein kinase (AMPK)/mTOR/survival signaling pathway (Jin et al., 2007). AMPK is an enzyme that is essential for maintaining the energy balance of cells. Its persistent activation can effectively slow down the cell proliferation rate and trigger the apoptotic cell death program (Xiang et al., 2004; Zheng et al., 2012). In addition, deguelin can also induce apoptosis of gastric cancer cells through the activation of two key apoptotic pathways, caspase-9 and caspase-3 (Lee et al., 2010)

### 3.2 Antiangiogenic and antimetastatic effects

The formation of neovascularization and tumor migration are indispensable key links for tumor cell growth and survival, and they are directly related to the prognosis and survival rate of cancer patients. Metastasis of cancer cells, as one of its malignant characteristics, greatly increases the difficulty of treatment and leads to higher mortality. Tumor metastasis is a complex and multistage process, including the invasion and migration of cancer cells into the circulatory system and lymphatic system, followed by angiogenesis and extravasation to distal tissues and organs (Aggarwal et al., 2020a; Aggarwal et al., 2020b; Aggarwal et al., 2019). As a potential therapeutic molecule, deguelin can inhibit angiogenesis and tumor metastasis through a variety of targets, such as MMPs, HIF-1α, FAK and PI3K/Akt pathway (Nguyen et al., 2015). In addition, deguelin can also act on a variety of angiogenesis related proteins, such as VEGF, VEGFR and PECAM-1, showing significant anti angiogenesis and anti metastasis effects (Lee et al., 2010).

Tumor cells achieve invasion and metastasis through the expression and activation of proteases, enabling them to breach the extracellular matrix (ECM). Matrix metalloproteinases (MMPs) are the primary enzymes responsible for ECM degradation in humans and play a crucial role in tumor initiation and progression. Tumor cells secrete various MMPs, among which MMP-2 and MMP-9 can degrade nearly all types of ECM components. By disrupting the histological barriers to tumor cell invasion and activating other MMPs, they trigger a cascade reaction that collectively promotes tumor cell invasion and metastasis. Several studies have shown that deguelin effectively inhibited the migration and invasion of triple negative breast cancer and human osteosarcoma cells by downregulating MMP-2 and Janus kinase 2 (JAK2)/STAT3 signaling pathways (Cho et al., 2019; Shang et al., 2014). In the study of mouse breast cancer cell lines, deguelin also showed the ability to inhibit cell migration and invasion by targeting PI3K/Akt signaling pathway (Mehta R., et al., 2013). In human hepatocellular carcinoma, deguelin exerts potent anti angiogenic effects by specifically acting on the hepatocyte growth factor receptor (HGF-c-Met) and VEGF-VEGFR pathways in cancer cells and endothelial cells, respectively (Li, Yu, Li, et al., 2018). For head and neck squamous cell carcinoma and non-small cell lung cancer, deguelin significantly reduced the migration ability of cells by targeting epidermal growth factor receptor (EGFR) and its downstream PI3K/Akt and mitogen activated protein kinase/extracellular signal regulated kinase (MEK/ERK) signaling pathways (Baba et al., 2015; Baba et al., 2017; Gao et al., 2020; Mittal et al., 2020). It is worth noting that in the non-small cell lung cancer (NSCLC) mouse model, deguelin was found to inhibit the EMT process by promoting the expression of PTEN and KLF4, which further weakened the invasion and migration ability of NSCLC cells (Lu et al., 2022). In addition, deguelin can inhibit EMT and metastasis of non-small cell lung cancer cells by inactivating NIMA related kinase 2 (Zhao et al., 2017). In lung cancer cells, deguelin can also reduce the expression levels of MMP, p-ERK1/2, p-Akt (Thr308), Ras homology family member A (Rho A), p-p38, p-JNK and NF-κB (p65) and other molecules (Hsiao et al., 2018). It is worth mentioning that deguelin mediated inhibition of MMP-2 and MMP-9 has also been reported in pancreatic cancer (Zheng et al., 2016).

### 3.3 The synergistic effect of deguelin in cancer

Deguelin exerts its anticancer potential in solid tumors by inducing tumor cell death (Tables 1, 2). Its combination with conventional chemotherapy has also been shown to enhance the efficacy (Ranjan et al., 2015). Bae, S., et al. revealed that deguelin effectively reversed the resistance of SKOV3 ovarian cancer cells to paclitaxel by downregulating EGFR and its downstream signaling pathways, and regulating the expression of Bcl-2 family proteins (Bae et al., 2024). In addition, deguelin can inhibit the autophagy process of pancreatic cancer cells, and significantly enhance the anticancer effect of doxorubicin on pancreatic cancer cells through chemical sensitization (Xu et al., 2017). In MGC-803 gastric cancer cells, the combination of deguelin and cisplatin also showed significant proliferation inhibition (Li et al., 2014). On the other hand, deguelin performed well in enhancing the apoptosis inducing activity of EGFR tyrosine kinase inhibitor AG1478 on mutant head and neck squamous cell carcinoma (Baba et al., 2017). Similar findings have also been reported in breast cancer (Robles et al., 2016), B-cell chronic lymphocytic leukemia (Rebolleda et al., 2016), lung cancer (Kim et al., 2009) and esophageal squamous cell carcinoma (Baba and Kato, 2017). It is particularly worth mentioning that at similar median inhibitory concentration (IC_50_), the combined administration of deguelin and docetaxel not only significantly inhibited the formation of spheroids and the migration ability of tumor cells, but also induced the apoptosis of tumor cells (64). These research results fully demonstrate the anticancer potential of deguelin in combination chemotherapy, but more research is still needed to further verify its synergistic anticancer effect.

**TABLE 1:** Anticancer effects and molecular mechanisms of action of deguelin based on *in vitro* studies.

Cancer type	Cell lines	Mechanisms and effects	References
Non-small cell lung cancer	A549 and H1299	Inhibit PI3K/Akt signaling pathway and trigger mitochondrial mediated apoptosis pathway. The cell cycle was arrested in G2/M phase to inhibit the migration and invasion of these 2 cells.The inhibitory effect exhibited both concentration- and time-dependent characteristics. The IC50 values (μM) for the A549 cell line at 24, 48, and 72 h were 10.32 ± 1.21, 7.11 ± 0.82, and 5.55 ± 0.42, respectively; while for the H1299 cell line, the corresponding IC50 values (μM) at 24, 48, and 72 h were 5.95 ± 0.60, 2.05 ± 0.18, and 0.58 ± 0.23, respectively.	Wang et al. (2018)
	NCI-H520 and SK-MES-1	Inhibit angiogenesis and metastasis of non-small cell lung cancer cells by inactivating NIMA related kinase 2.	Zhao et al. (2017)
Lung adenocarcinoma	A549	By acting on Slit2/Robo2 and downstream targets, it reduces the expression of MMP, thereby affecting the quantity and morphology of cytoskeletal protein F-actin. Ultimately, this leads to a decrease in cellular proliferation, invasion, and migration abilities.	Zhou H. et al. (2020)
Lung squamous cell carcinoma	NCI-H520 and SK-MES-1	Downregulate the expression of Galectin-1 in a time- and concentration-dependent manner, subsequently inhibiting the MAPK signaling pathway and inducing apoptosis in lung squamous cell carcinoma cells.	Yan et al. (2016)
Breast Cancer	MCF-7	Downregulation of anti-apoptotic signaling pathways such as PI3K/Akt effectively induced apoptosis in cancer cells.	Chu et al. (2011)
	4T1	Inhibit cell migration and invasion by targeting pi3k/akt signaling pathway.	Mehta R. R. et al. (2013)
Ovarian cancer	SKOV3	Upregulate the expression of KLF15, inhibit cell proliferation, and induce apoptosis.	Zhao et al. (2022)
Multiple myeloma cells	U266	Regulate Akt and p38 MAPK signaling pathways, affect the mRNA expression that regulates the cell cycle, induce apoptosis and arrest cells at the G2/M mitotic stage, thereby significantly reducing the growth, migration and invasion ability of cells.Deguelin exhibited an IC50 of 6 μmol/L against U266 cells.	Sun et al. (2024)
Carcinoma of gallbladder	GBC-SD and SGC-996	Inhibit PTEN/PI3K/Akt signaling pathway and reverse EMT, and inhibit the proliferation, migration and invasion of gallbladder cancer cells in a dose-dependent manner.	Wang (2020)
Prostate cancer	LNCaP and PC-3	Downregulate the phosphorylation levels of Akt and GSK-3 β, promote the protein degradation of β -catenin, reduce its accumulation in the nucleus, and then inhibit the transcriptional activation of β -catenin non responsive genes, thus effectively inhibiting the growth, proliferation, invasion and migration of prostate cancer cells. It can also inhibit the expression of proliferation related proteins and anti-apoptotic proteins in prostate cancer cells.	Fang et al. (2024); Thamilselvan et al. (2011)
Gastric cancer	MGC-803and MKN-45; SGC7901/VCR	Apoptosis of gastric cancer cells is induced by the activation of two key apoptotic pathways, caspase-9 and caspase-3.Deguelin demonstrated IC50 values of 11.83μM and 9.33 μM in MGC-803 and MKN-45 cells, respectively, after 72 h of treatment.	Kang et al. (2018); Lin et al. (2021); Lz et al. (2018)
GBM	GBM C6; DBTRG; GL261; Human umbilical vascular endothelial cell (HUVECs);	Promote cell cycle arrest at G2/M stage and induce apoptosis, reduce its proliferation activity, and inhibit the migration of GBM cells. Inhibiting the CCL2/NF-κB pathway *in vivo* to curb tumor growth and inhibit GBM angiogenesis.The IC50 of deguelin on DBTRG cells after 24 h was 4.178 μM, and the IC50 of deguelin on C6 cells after 12 h was 1.953 μM.	Qian et al. (2024)
Colon cancer	HT-29	Arrest cells in G1-S phase.The IC50 value of deguelin against HT-29 colon cancer cells was 4.32*10-8M.	Kang et al. (2012)
Esophageal squamous cell carcinoma	Eca109,KYSE180 and KYSE450; KYSE150;	It plays an important role in inhibiting the activity of Aurora B kinase, which is necessary to promote cell mitosis.	Baba and Kato (2017); Yu et al. (2017)
Triple negative breast cancer and human osteosarcoma cells	U-2 OS	Downregulate MMP-2 and JAK2/STAT3 signaling pathway, inhibit its migration and invasion ability.	Cho et al. (2019); Shang et al. (2014)
Liver cancer	HUVECs/a human hepatocellular patient-derived xenografts (PDXs) tumor model;	It specifically targets the HGF-c-Met and VEGF-VEGFR pathways in cancer cells and endothelial cells, exerting potent anti-angiogenic effects.	Li et al. (2018)
Head and neck squamous cell carcinoma and non-small cell lung cancer	Ca9-22 and HSC-4; SCC-4 and HSC-4;	By targeting EGFR and its downstream signaling pathways such as pi3k/akt and mek/erk, the cell migration ability was significantly reduced.	Baba et al. (2015); Baba et al. (2017); Gao et al. (2020); Mittal et al. (2020)

**TABLE 2:** Anti-cancer effects and molecular mechanisms of action of deguelin based on *in vivo* studies.

Cancer types	Animal models	Mechanisms	Effects	Dosage and Duration	References
Head and neck squamous cell carcinoma	Female BALB/cnude mice injected with Hep-2 cells	Induce apoptosis and autophagy in HNSCC cells by regulating multiple signaling pathways.	Inhibit tumor growth	4 mg/kg and 3weeks	Yang et al. (2013)
Lymphocytic leukemia	Female young NZB mice (NZB/Ola Hsd) transplanted with spleen cells from aged NZB mice with lymphoproliferation	Downregulate AKT, NF-κB and several downstream anti-apoptotic proteins, activating the mitochondrial pathway of apoptosis. Inhibit stromal cell-mediated c-Myc upregulation and resistance to fludarabine, increasing fludarabine induced DNA damage.	Prolong the survival of transplanted mice	4 mg/kg deguelin and/or 35 mg/kg fludarabine and 61 days	Rebolleda et al. (2016)
Oral	NOD-SCID female mice intrabuccally transplanted with SAS-GL cells	Suppress the invasion and migration of oral cancer by downregulating TNF-α-induced NF-κB signaling.	Inhibit metastasis	0.8 and 4.0 mg/kg and14 days	Liu et al. (2016)
Colon	Female nude Mice xenografted with SW620 cells	Inhibit CRC cell growth by inducing apoptosis *via* activation of p38 MAPK pathway.In addition, the IC50 value of deguelin in SW-620 colon cancer cell line was 4.62*10^−7^M。	Decrease tumor growth	4 mg/kg and24 days	Chen et al. (2019)
Breast	BALB/c female mice injected with 4T1 cells	Mediated by AKT and ERK mediated signaling pathways.	Prevent metastasis	2 mg/kg and 6 mg/kg and 20 days	Mehta R. R. et al. (2013)
	Female athymic mice (nu/nu) xenografted with MDA-MB-231	Mediated through EGFR-PAKT/c-Met p-ERK and NF-κB by down regulating their downstream targets such as p-STAT3, c-Myc, Survivin.	Reduce tumor growth	2 or 4 mg/kg and 21 days	Mehta R., et al. (2013)
Lung	Female athymic nude mice xenografted with HCC827 cells, H1975, A549 and H3255 cells	Directly down-regulating of EGFR-signaling pathway.	Reduce tumor size	3 mg/kg and 32–41 days	Gao et al. (2020)
	Athymic nude mice injected with A549 cells	Anti-metastatic effect on the suppression of CtsZ signaling.	Inhibit metastasis	4 mg/kg and 28 days	Li, Yu, Ma, et al. (2018)
	Athymic nude mice xenografted with NCI-H1299 or A549 cells	Inhibit the growth of NSCLC cells both *in vitro* and *in vivo* by downregulation of Bmi1 expression.	Inhibit tumor growth	3 mg/kg and 21 days	Li, Yu, Xia, et al. (2018)
	Female nude mice xenografted with A549 cells	Induce cell death of LCCs by triggering expression of PUMA.	Stimulate cell death	1 mg/kg DOX, 2 mg/kg deguelin and 18 days	Wang and Wang (2018)
	Six-week-old female BALB/c-nude mice	Inhibit tumor growth and upregulate the expressions of PTEN and KLF4 in tumor tissues.	Inhibit tumor growth	0.9%NaCl,4 mg/kg deguelin, Gavage and 2 weeks	Hsiao et al. (2018)
Hepatocellular	Female athymic nude mice Xenografted with HepG2 cells	Inhibit HCC through suppression of angiogenesis on vascular endothelial cells and reduction of proangiogenic factors in cancer cells.	Delay tumor growth	4 mg/kg and 34 days	Li et al. (2018)
Pancreatic	Athymic nude mice orthotopically implanted with PanC-1-luc cells	Target NF-κB to induce reversal of EMT and apoptosis.In addition, the IC50 (24 h) value of deguelin against PanC-1 cells was 62 mM.	Decrease tumor growth	5 mg/kg and 37 days	Boreddy and Srivastava (2013)
GBM	C57BL/6 male mice/inject GL261 cells to establish a syngeneic orthotopic GL261 GBM model	Inhibit the ccl2/nf- κ B pathway *in vivo* to curb tumor growth and inhibit GBM angiogenesis.	Inhibit tumor growth and inhibit GBM angiogenesis.	Inject with saline or deguelin	Qian et al. (2024)

Deguelin also showed unique advantages in radiotherapy sensitization. The abnormally high expression of fbxo22 in lung cancer was confirmed as a new molecular marker of radioresistance and a biomarker of poor prognosis. Silencing fbxo22 would increase the radiosensitivity of lung cancer (Zhang et al., 2019). Mechanistically, fbxo22 promotes the transcription of Rad51 gene by increasing the level of FoxM1 at the Rad51 promoter, thereby reducing the sensitivity of lung cancer cells to radiotherapy (Li et al., 2021). Therefore, drug targeted therapy targeting fbxo22 is expected to become a new strategy to improve the efficacy of radiotherapy. Deguelin was able to induce gene expression profile changes similar to those after fbxo22 silencing, resulting in the downregulation of homologous recombination (HR) molecules, especially Rad51. Overexpression of Rad51 could reverse the radiosensitization effect mediated by degumming protein. Importantly, knockdown of fbxo22 significantly reduced the responsiveness of lung cancer cells to deguelin, suggesting that the mechanism of deguelin action is dependent on the fbxo22/rad51 pathway. Deguelin was identified as a small molecule inhibitor of fbxo22, which can enhance the sensitivity of lung cancer radiotherapy *in vitro* and *in vivo*, and has good safety, with rich prospects for clinical transformation (Chen et al., 2024).

## 4 The novel design of deguelin derivatives

Heat-shock proteins (HSPs), functioning as molecular chaperones, are ubiquitously present proteins. HSP90, a heat-shock protein, plays a pivotal role in maintaining the stability and activation of numerous client proteins involved in cellular signal transduction pathways, relying on its molecular chaperone function (Whitesell and Lindquist, 2005). Structurally, HSP90 consists of three main domains: the N-terminal ATP-binding domain (NTD), the middle co-chaperone and client-binding domain (MD), and the C-terminal dimerization domain (CTD). The NTD features an adenine-nucleotide-binding pocket, and this ATP-binding region holds promise for the discovery of highly selective HSP90 inhibitors (Stebbins et al., 1997). Moreover, HSP90’s molecular interactions are greatly facilitated by the C-terminal ATP-binding domain. Notably, experimental evidence has demonstrated that deguelin does not bind to the ATP-binding pocket within the N-terminal domain of HSP90. Instead, it may bind to alternative binding sites on HSP90, thereby differentiating itself from other HSP90 inhibitors (Zhang et al., 2010). Despite several studies showing deguelin’s inhibitory effect against HSP90, the precise binding site of deguelin on HSP90 is still being investigated (Oh et al., 2007).

**FIGURE 5:**
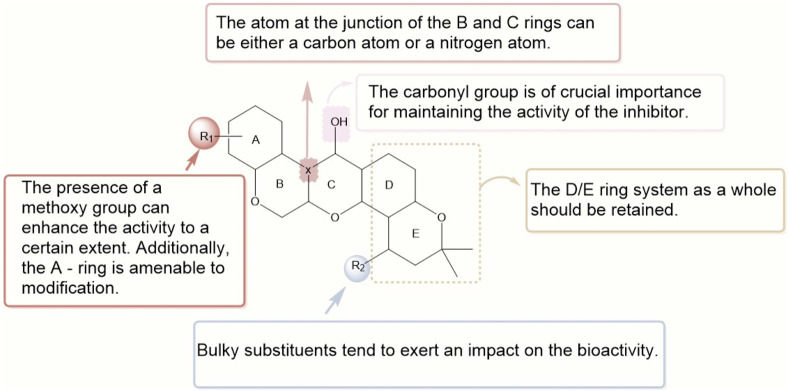
The relationship between structure and activity in deguelin and its derivatives.

The synthesis of deguelin is exceedingly challenging due to restricted resources and a complicated molecular architecture. Furthermore, deguelin can elicit a state resembling Parkinson’s disease, characterized by a reduction in tyrosine hydroxylase immunoreactivity in the rat brain. Long-term or high-dose deguelin administration induces a Parkinson’s disease-like condition in rats (Caboni et al., 2004), characterized by a decrease in tyrosine hydroxylase-positive neurons. These issues considerably hinder the use and advancement of deguelin. Consequently, there is a growing demand for novel compounds that are either more efficacious or less harmful than deguelin. Deguelin comprises five contiguous rings, creating a distinctive stiff pentacyclic configuration. Direct interaction with active compounds is significantly impeded by steric hindrance. The SARs of deguelin are illustrated in Figure 5. The methoxy groups at C9 and C10, in conjunction with the ketone carbonyl group at C7, have been recognized as essential binding sites for HSP90 (Kim et al., 2008). Altering these places signifies the primary focus of adjustment. The most prevalent derivatives of deguelin are B/C-ring-truncated and C-ring-truncated. Consequently, alterations to the A/B rings and substitution of the linker are executed.

### 4.1 Modification and antitumor activity of B/C-ring derivatives of deguelin

#### 4.1.1 C - Mediated truncation of the B/C-ring structure

##### 4.1.1.1 The main modification of the linker

**FIGURE 6:**
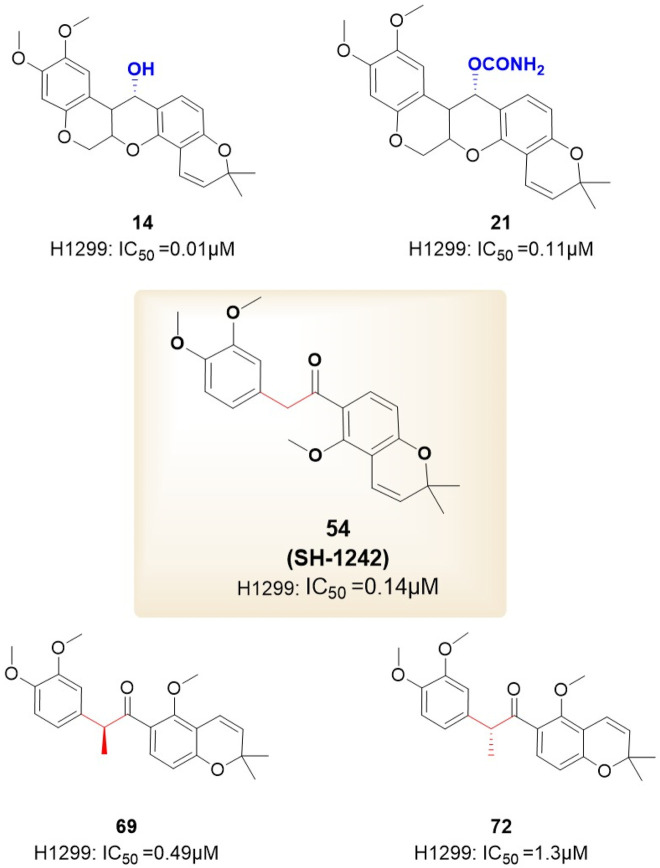
Novel B/C-ring-truncated deguelin derivatives **54** (SH-1242) and various analogs featuring diverse linker designs.

In 2012, Chang et al. (2012) introduced the BC-ring truncation technique, which explored the SAR of deguelin and delineated the modification trajectory for subsequent alterations of deguelin (Figure 6).

They developed 24 analogs with alterations to the methoxy groups on the A-ring, BC-ring, and E-ring. The findings indicated that the methoxy groups at C9 and C10 of the A-ring, the cis conformation of deguelin, and the existence of the C7 carbonyl group were crucial for preserving deguelin’s activity. Furthermore, the ring-opening of the terminal 2,2-dimethyl-2H-chromene moiety resulted solely in a reduction or complete loss of activity. Consequent on these findings, the study emphasis transitioned to nine B- and/or C-ring truncated molecules. The truncated compounds without the B or C ring exhibited markedly different activities relative to deguelin, suggesting that the removal of either the B or C ring was suboptimal. In contrast, the elimination of both the B- and C-rings was advantageous for augmenting inhibitor efficacy. Furthermore, methoxy groups were crucial in the SAR. Compound **54**, featuring methoxy groups (IC_50_ = 140 nM), demonstrated inhibitory action comparable to that of deguelin. Nonetheless, following the substitution of the methoxy groups, the activity diminished by a factor of 10. The enantiomers of deguelin analogs demonstrated distinct activity patterns. The (S) enantiomer exhibited an IC_50_ of 490 nM, indicating nearly double the potency of the racemate (IC_50_ = 730 nM). The (R) enantiomer exhibited an IC_50_ of 1,300 nM, demonstrating almost half the activity of the racemate. This occurrence underscores the importance of stereochemistry in the efficacy of HSP90 inhibitors. Unexpectedly, these inhibitor compounds exhibiting remarkable inhibitory efficacy demonstrated a direct association between cell growth inhibition and HIF-1α suppression. Among the compounds, analog **14** (IC_50_ = 10 nM) demonstrated the most powerful suppression of HIF-1α, surpassing the inhibition rate of deguelin. Furthermore, analogs **54** (IC_50_ = 140 nM) and 69 (IC_50_ = 490 nM) inhibited the interaction between HIF-1α and HSP90 *in vitro*. The results strongly indicate that analogs **54** and **69** may impede HSP90 activity by competing with ATP at the ATP-binding site. *In vitro* investigations on zebrafish embryos further validated that the antiangiogenic efficacy of analog **69** appeared superior to that of **54**.

Nonetheless, the anti-angiogenic mechanisms of analogs **54** and **69** require additional examination.

**FIGURE 7:**
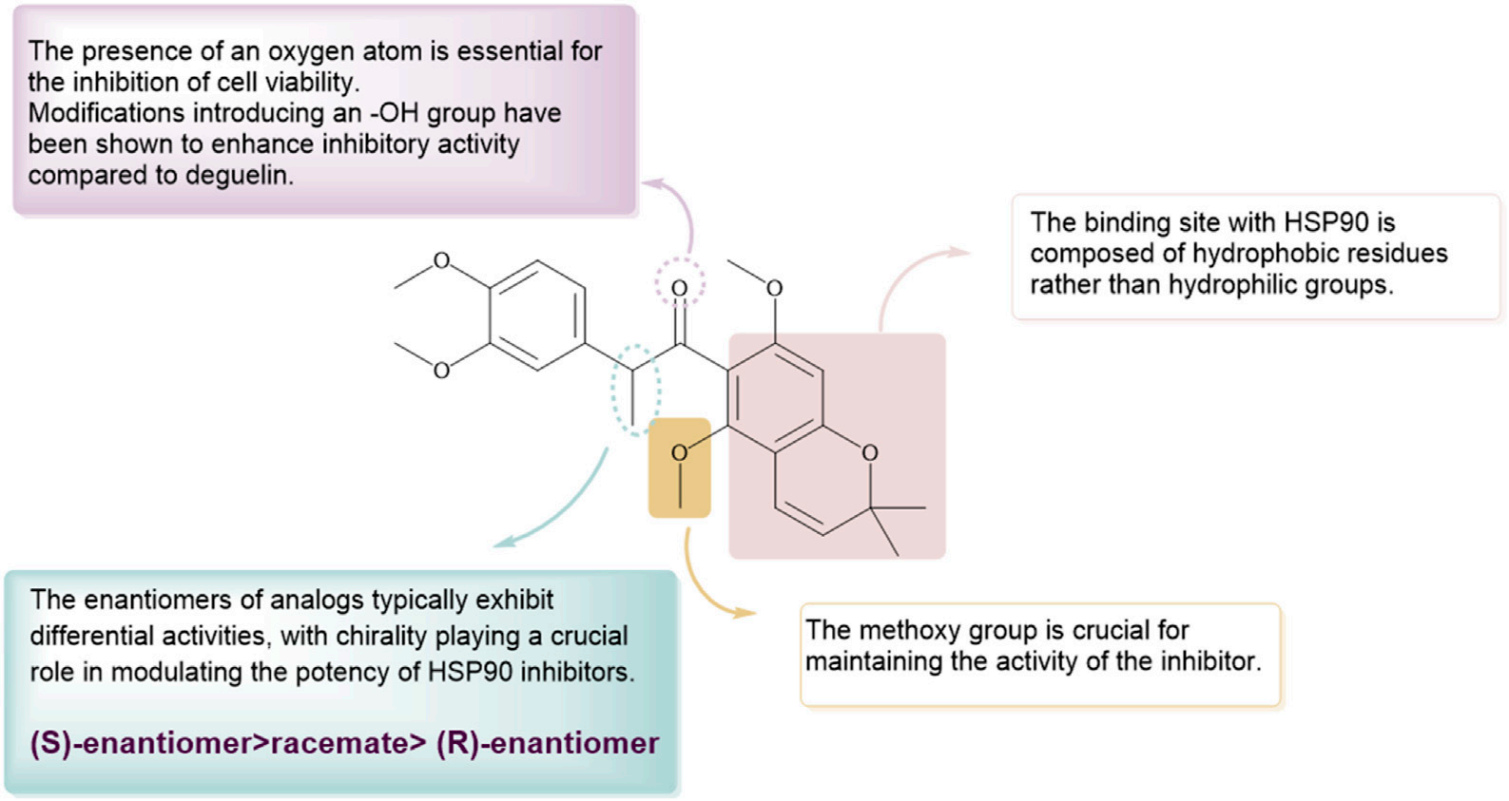
The SAR of SH-1242 and its analogs.

In the following year, the therapeutic potential of compound **54**, referred to as SH-1242 (Lee et al., 2016), was further examined. This research employed various cancer sublines (designated as “/R”) exhibiting acquired resistance to anticancer therapies, tackling the issue of drug resistance encountered in the clinical use of anticancer agents (Figure 7). SH-1242 markedly reduced the viability of both drug-sensitive and drug-resistant sublines, demonstrating notable potency in PC-9 cells (IC_50_ = 800 nM) and effectively inducing apoptosis in these cells. The findings align with results from *in vitro* experiments on human NSCLC cell-derived xenograft tumors. SH-1242 exhibited antitumor activity similar to deguelin and geldanamycin, with no detectable toxic effects on major organs such as the liver, lungs, heart, kidneys, spleen, bladder, ovaries, stomach, pancreas, colon, and rectum. Additionally, SH-1242 markedly inhibited the proliferation of lung tumors driven by mutant KRAS. In contrast to deguelin, which has been linked to parkinsonism-like syndrome, SH-1242 demonstrated significantly reduced cytotoxicity towards normal cells, including hippocampal neurons and retinal pigment epithelial cells, and had negligible effects on tyrosine hydroxylase immunoreactivity in the substantia nigra of rats. SH-1242 binds to both the N-terminal and C-terminal domains of Hsp90, primarily acting through direct interaction with the C-terminal ATP-binding site. The binding interfered with Hsp90 chaperone activity, identifying residues K615 and S677 in the C-terminal domain as essential for ATP binding. The results collectively highlight the efficacy and safety of SH-1242, positioning it as a promising candidate for the development of Hsp90 C-terminal inhibitors and providing substantial evidence for its potential as a therapeutic agent.

**FIGURE 8:**
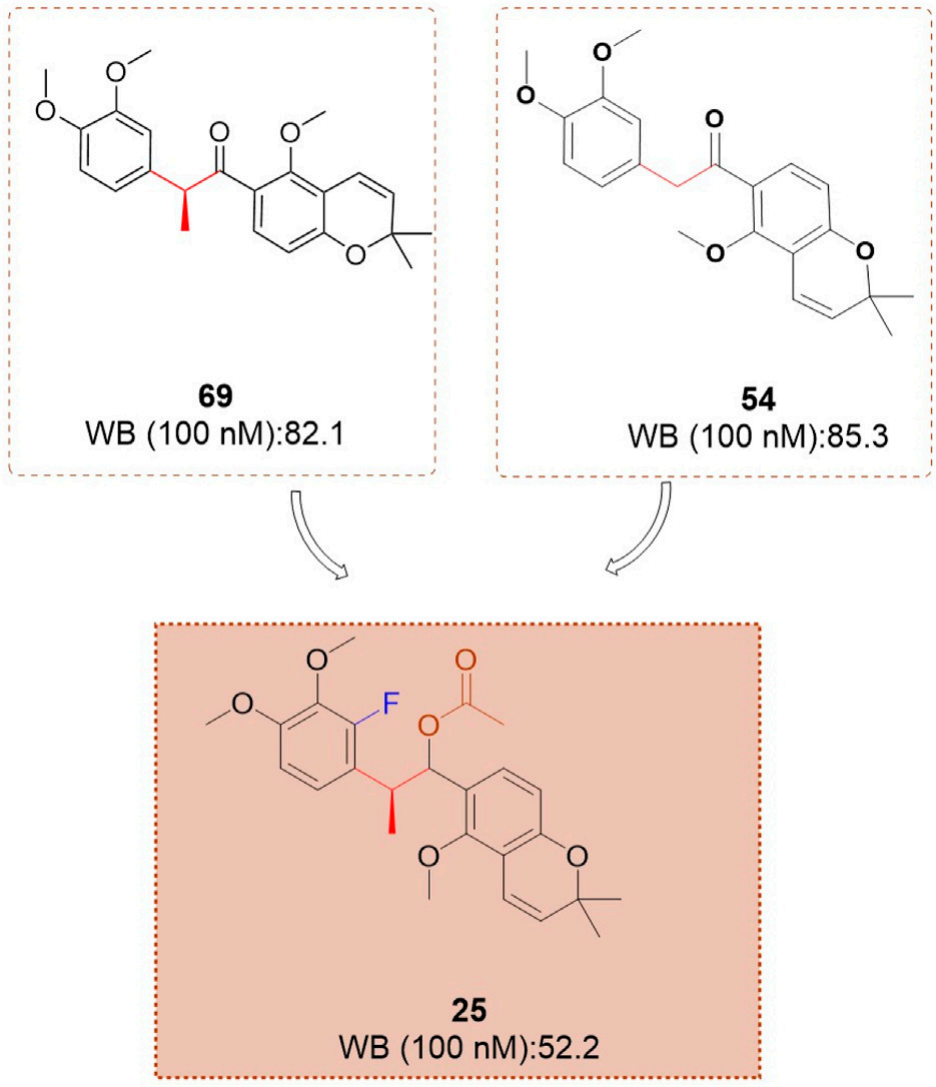
The structure of compound **25**.

Based on investigations of compounds 69 and SH-1242, Kim et al. (2015) have developed a series of deguelin derivatives that exhibit both enhanced efficacy and reduced toxicity. In these analogues, the α-methylcarbonyl linker was replaced with various bioisosteres—including olefins, diols, alcohols, and acyl groups—to probe structure–activity relationships. Notably, the methyl-oxime modification within the pyridine A-region (compound 51) afforded substantially greater inhibitory potency than deguelin itself. Moreover, among the 7-carbonyl oxime derivatives (and their reduced and further acylated congeners), the E-configured isomers consistently outperformed their Z counterparts, each retaining an adjacent α-methyl group; this substituent appears to impose a conformation that is particularly favorable for HIF-1α suppression. Ultimately, compound **25 (** Figure 8) demonstrated robust antiproliferative activity in human non–small cell lung cancer H1299 cells and potently inhibited hypoxia-induced angiogenesis in human retinal microvascular endothelial cells. Molecular docking studies confirmed that compound 25 occupies the ATP-binding pocket of Hsp90’s C-terminal domain, thereby destabilizing HIF-1α and mediating its antitumor effects.

##### 4.1.1.2 The derivatization of the ring

**FIGURE 9:**
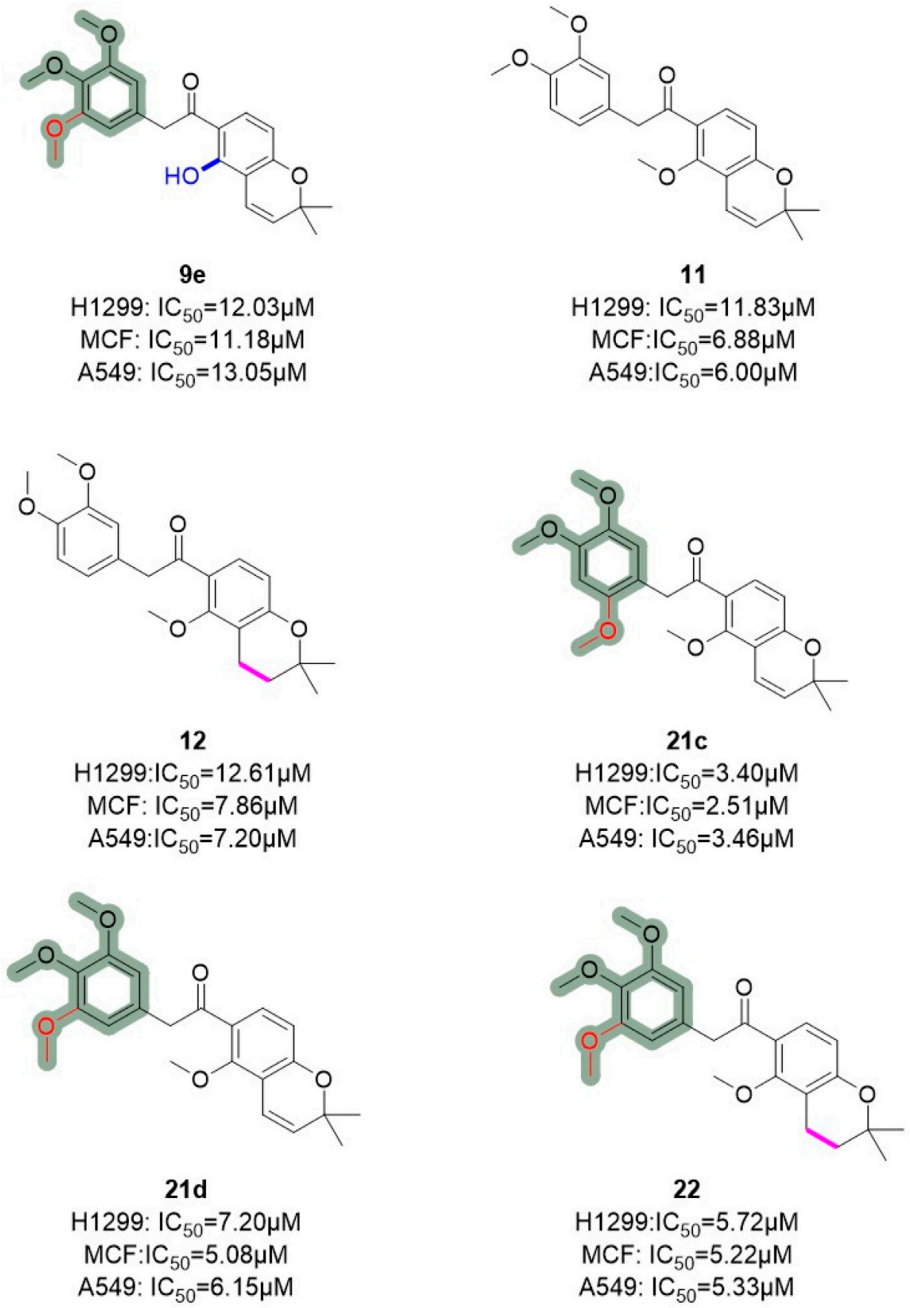
Novel B/C-ring-truncated deguelin derivatives with A -ring modifications.

**FIGURE 10:**
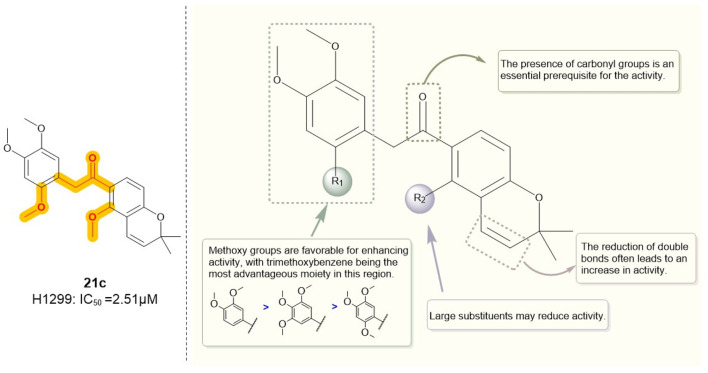
Figure The SARs of **21c** and its analogs.

Similarly, Hong et al. (Yao et al., 2019) employed truncation strategies to optimize the B and C rings of deguelin derivatives. The design and synthesis of 24 B- and C-ring-truncated derivatives led to the identification of a novel Hsp90 inhibitor with promising potential (Figure 9). The structural modifications preserved the D- and E-ring backbone of deguelin while altering R2 and the carbonyl group. In contrast to the methodology employed by Chang et al., they conducted a more detailed examination of the influence of the number and position of methoxy groups in the A ring on antitumor efficacy. Compounds containing trimethoxy phenyl groups (**9e**, **21c**, **21d**, and **22**) demonstrated increased inhibition rates against human non-small cell lung cancer H1299 cells. In contrast, substituting methoxy groups with chlorine atoms led to a marked decrease in antiproliferative activity. Modification of the D/E ring revealed that large substituents on the D ring significantly diminished activity, whereas hydroxyl and methoxy groups increased it. Additionally, a decrease in the number of double bonds in the E ring enhanced the activity. Compound 21c, which contains a 2,4,5-trimethoxyphenyl group, exhibited significant inhibitory activity against Hsp90 (IC_50_ = 60 nM) and showed a threefold enhancement in the inhibition of MCF-7 cells relative to the parent compound deguelin (IC_50_ = 251 nM). Experimental data demonstrated that 21c induces apoptosis in MCF-7 cells, as indicated by chromatin condensation, phosphatidylserine externalization, reduced Bcl-xL levels, and elevated cleaved caspase-3 expression. The findings highlight the considerable anticancer efficacy of **21c** (Figure 10), positioning it as a promising candidate for the advancement of Hsp90 inhibitors.

**FIGURE 11:**
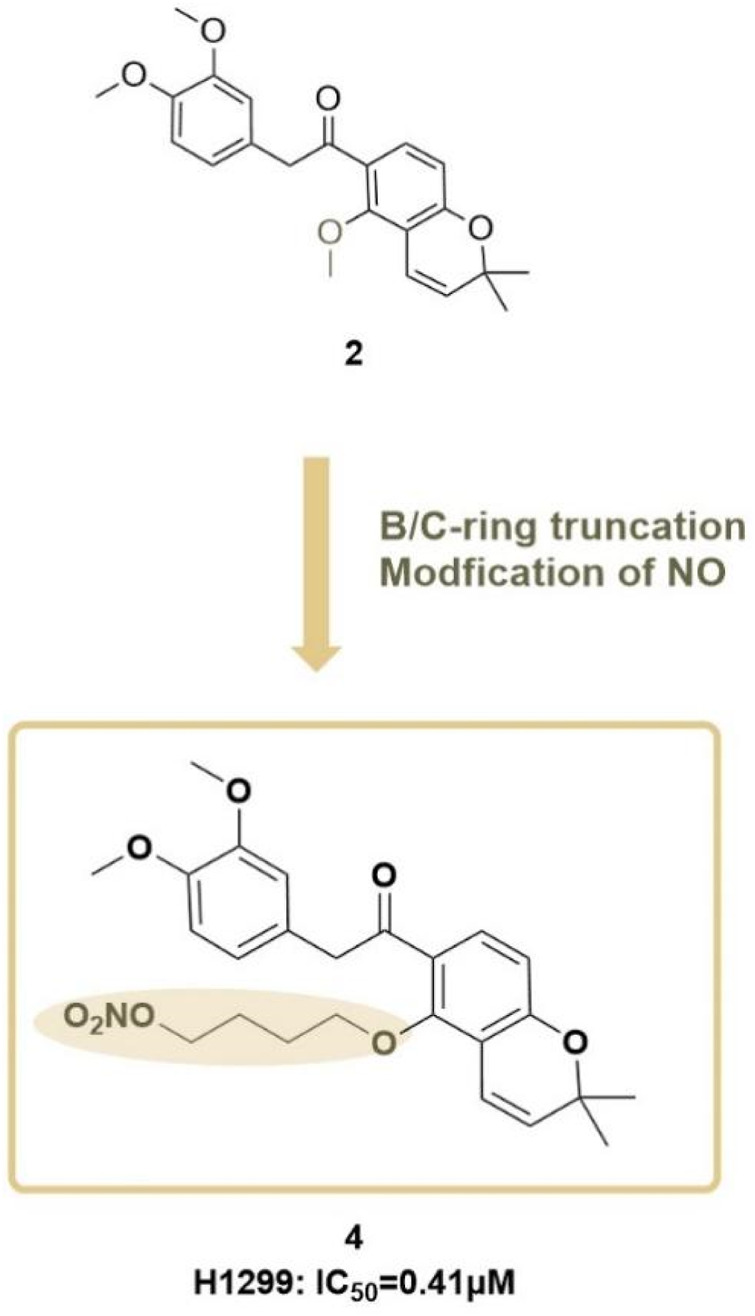
Design strategy for B/C- ring-truncated NO derivatives of deguelin.

Following this, Zhou H. et al. (2020) from the same research group proposed a new structural modification strategy. It was noted that while the B/C-ring deguelin analogue compound **2** (referred to as compound **12**) exhibited anticancer activity, its comparatively low potency limited its therapeutic applicability in lung cancer treatment. The team sought to develop novel deguelin derivatives to improve anticancer efficacy. They designed and synthesized a novel hybrid compound **4** (Figure 11), incorporating a nitric oxide (NO) donor moiety, with the expectation of enhancing its anticancer activity and therapeutic utility in lung cancer treatment.

In antiproliferative assays involving three lung cancer cell lines (A549, H596, and H1299), compound 4 demonstrated significantly greater inhibitory effects than both deguelin and compound **2**. In the H1299 cell line, compound 4 demonstrated significant antiproliferative activity, indicating it is approximately 7-fold and 55-fold more potent than compound **2** and deguelin, respectively. Additionally, compound **4** demonstrated an inhibitory effect on the growth of H1299 cells, which was both time- and dose-dependent. The presence of the NO scavenger PTIO diminished its antiproliferative activity, thereby confirming the essential role of NO in mediating its anticancer effects. Mechanistic investigations revealed that the nitrate moiety in hybrid **4** formed hydrogen bonds with GLY137, thereby effectively inhibiting Hsp90 function. The inhibition resulted in cell cycle arrest at the G2/M phase and markedly reduced the migration and invasion of lung cancer cells. The findings highlight the potential of this novel hybrid compound as a therapeutic agent for lung cancer, warranting further development and investigation.

#### 4.1.2 N - mediated truncation of the B/C - ring structure

**FIGURE 12:**
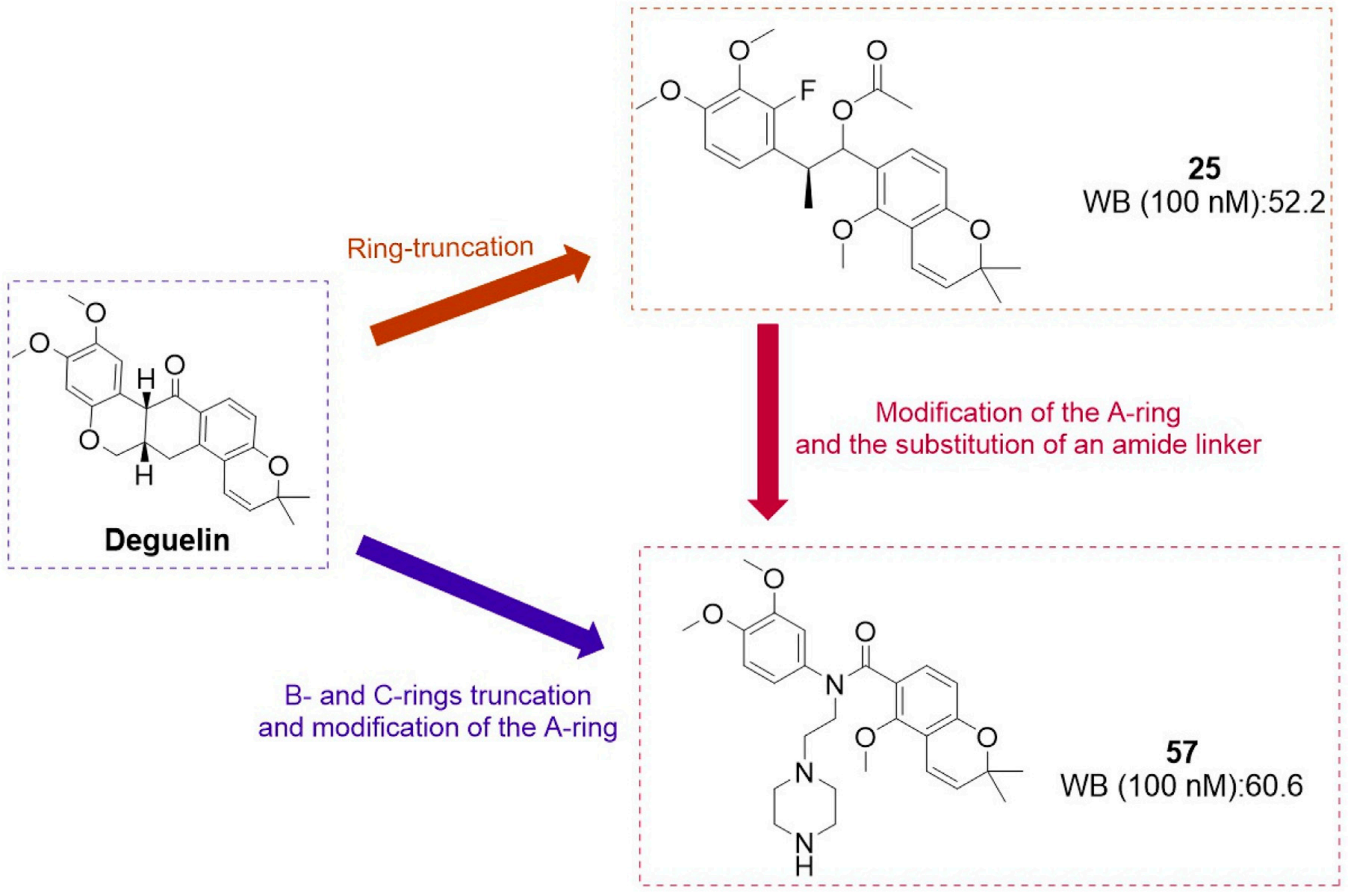
Design strategies for compounds **25** and **57**.

The research team focused on structural modifications of the A-ring and the linker of B- and C-rings to optimize compound **25** further. The A-ring was modified with 3,4-dimethoxyphenyl, fluorophenyl, or pyridine substituents, while the BC-ring linker was altered by substituting the C-C=O group with N-substituted amides (Kim et al., 2019). A series of deguelin analogues were synthesized, with the majority of pyridine-substituted A-ring derivatives demonstrating enhanced inhibitory activity relative to deguelin (Figure 12). The N-dimethyl and N-diethyl analogues consistently exhibited strong inhibitory effects. Certain N-benzyl analogues demonstrated significant inhibition, including 4-fluorobenzyl (63.1%), 4-chlorobenzyl (57.6%), and pyridin-4-yl (55.2%). Compound **57** demonstrated notable cytotoxicity at 10 μM against MDA-MB-231 (invasive ductal carcinoma) and 4T1 (murine breast cancer) cells, highlighting its potential as a promising candidate among these compounds. Exposure of MDA-MB-231 cells to compound **57** (2–10 μM) for 72 h resulted in significant morphological alterations, including cytoplasmic shrinkage. Mechanistic studies demonstrated that compound **57** inactivated MEK and reduced the expression and phosphorylation of critical HSP90 client proteins, specifically AKT, ERK, and STAT3. Molecular docking models indicate that compound **57** adopts a “T-shaped” conformation at the interface of the HSP90 homodimer, with the nitrogen atom in its piperazine ring forming a hydrogen bond with chain B. This interaction inhibits N-terminal dimerization and obstructs the formation of the ATP-binding pocket.

In conclusion, compound **57**is a promising candidate for the treatment of triple-negative breast cancer, acting as an HSP90 C-terminal inhibitor.

In contrast to the aforementioned studies, Nguyen et al. (2020) developed a series of deguelin derivatives featuring B/C ring-truncated scaffolds, specifically concentrating on A-ring substituted O analogues for structural modification. Various polar side chains were introduced as solubilizing moieties to the R group to enhance the pharmacodynamic and pharmacokinetic properties of these compounds. Cytotoxicity screening was conducted on two HER2-positive breast cancer cell lines, specifically trastuzumab-sensitive BT474 and trastuzumab-resistant JIMT-1, as well as normal human embryonic kidney (HEK293) cells. Nine compounds (**73**, **74**, **80**, **81**, **85**, **86**, **87**, **88**) significantly decreased the survival rates of BT474 and JIMT-1 cells to below 50%.

**FIGURE 13:**
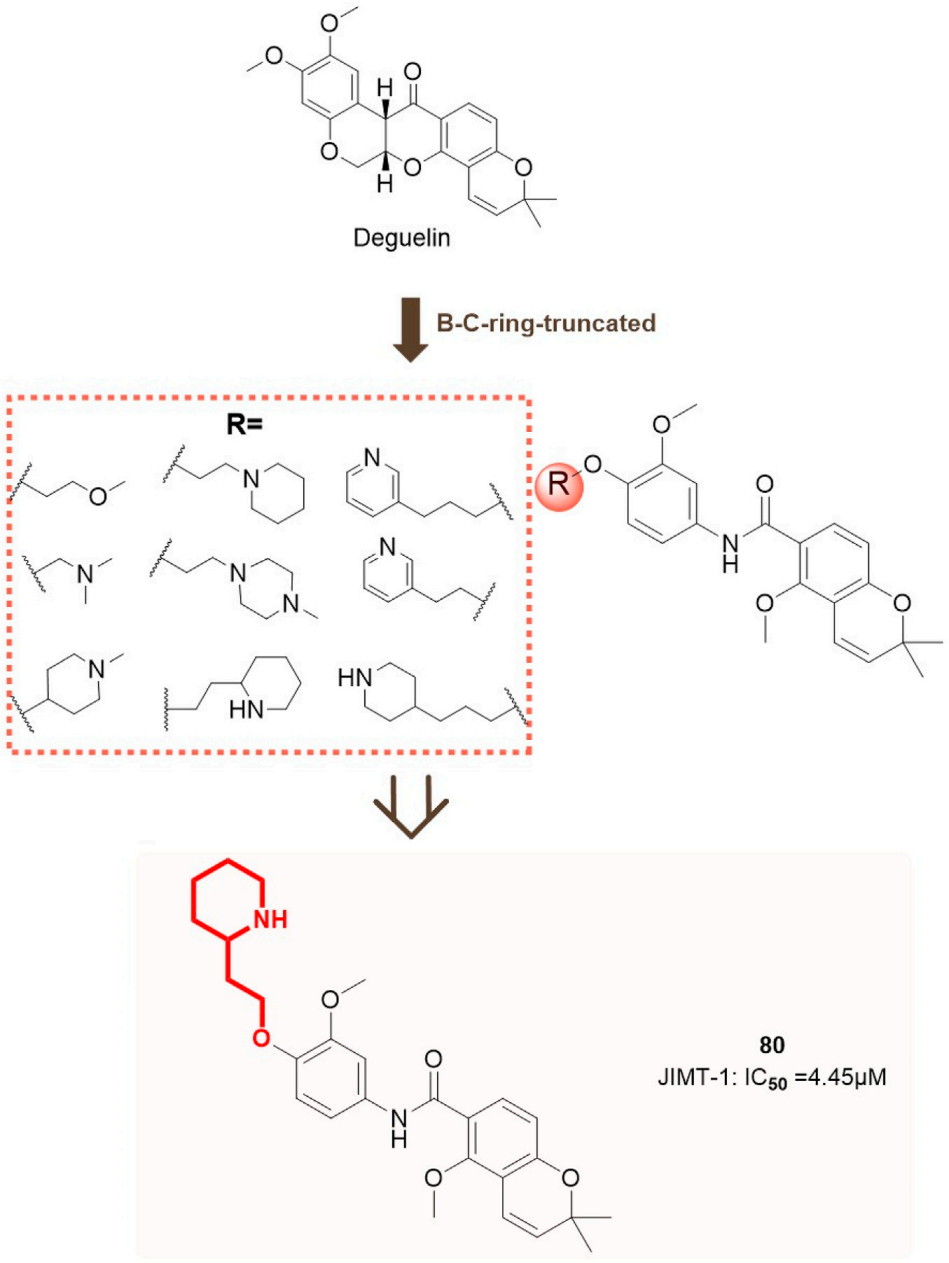
Design concepts for compound **80** and its analogues.

Compound **80** demonstrated the highest inhibitory activity against HER2-positive breast cancer cells, presenting IC_50_ values of 8.53 μM for BT474 cells and 4.45 μM for JIMT-1 cells, with no observed cytotoxicity towards normal HEK293 cells. Subsequent analysis indicated that compound **80** functions by binding to the C-terminal ATP-binding pocket of HSP90, thereby disrupting its interactions with chaperone and client proteins. The destabilization and inactivation of HSP90 client proteins occur without triggering the heat shock response (HSR), underscoring its selective mode of action. Molecular docking studies clarified the interaction mechanism of compound **80** with HSP90. The two-piperidine moiety of compound **80** occupies the ATP-binding pocket, where the deoxyribose moiety is located, establishing hydrogen bonds with Asn622 and Lys615 (chain A) and a π-cation interaction with Lys615. The interactions markedly improve the binding affinity of compound **80** for HSP90 (Figure 13), affirming its efficacy as a C-terminal HSP90 inhibitor.

**FIGURE 14:**
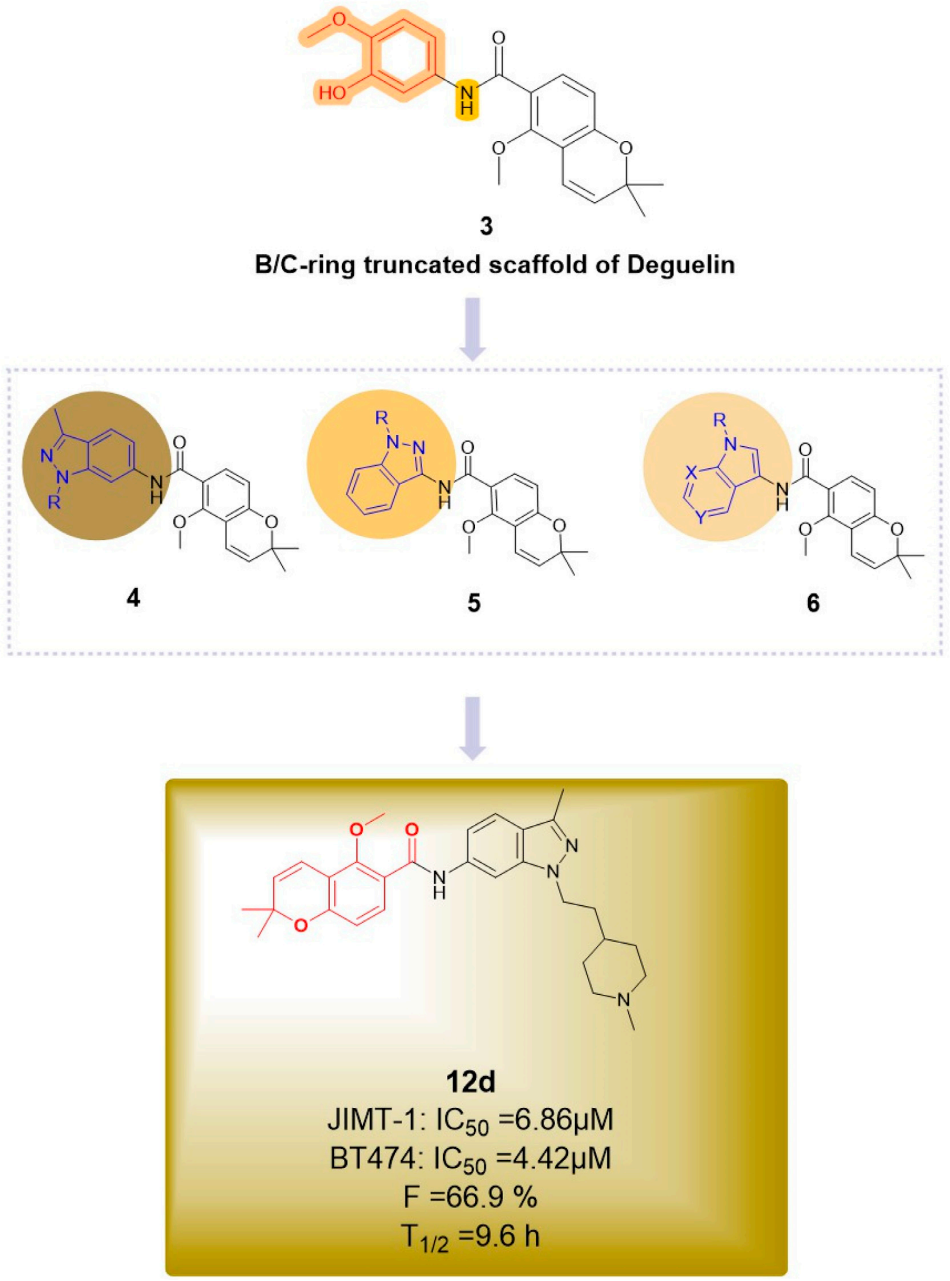
Novel deguelin B/C-ring-truncated derivatives derived from three indazole analogues.

The most recent report regarding B/C ring truncation is from La et al. (2024), who utilized compound **3** as a scaffold and replaced the dimethoxyphenyl group with an indole moiety to synthesize three indazole analogs. The antitumor activities of the indazole compounds were assessed in two HER2-positive breast cancer cell lines, BT474 and JIMT-1, as well as in normal human embryonic kidney (HEK293) cells. At a concentration of 10 μM, five compounds (**12d**, **12f**, **19c**, **19d**, **and 19g**) demonstrated significant inhibition of BT474 cell proliferation, with inhibition rates surpassing 50%. Compounds **12d** and **19c** exhibited comparable antiproliferative activity in JIMT-1 cells, demonstrating a dose-dependent reduction in phosphorylation levels in both trastuzumab-sensitive and -resistant cells. Compound **19c** was the only one to demonstrate cytotoxicity towards normal cells, influenced by the side chain, its position, and the ring structure. Compound **12d** (Figure 14) exhibited the highest inhibitory activity against HER2-positive breast cancer cells, with IC_50_ values of 6.86 μM for BT474 cells and 4.42 μM for JIMT-1 cells, while showing no significant impact on the proliferation of normal cells. *In vivo* experiments demonstrated that compound 12d inhibited tumor growth in trastuzumab-resistant xenograft mouse models. Additionally, compound **12d** demonstrated high oral bioavailability (F = 66.9%) and did not significantly inhibit hERG or CYP isoenzymes, while also yielding negative results in genetic toxicity assays. In comparison to the effective C-terminal inhibitor novobiocin, compound **12d** demonstrated enhanced therapeutic efficacy. The substantial inhibition of C-terminal HSP90 activity by **12d** is likely attributed to the robust π-cation interactions between the indazole and chromene aromatic rings and Lys615 in each hHSP90 chain, which exhibit near coplanarity. The protonated 1-methylpiperidine group is expected to localize to the ribose binding site within the ATP-binding pocket, potentially establishing a strong hydrogen bond with Glu611 in chain A. Consequently, compound 12d represents a viable candidate for further advancement as a C-terminal HSP90 inhibitor in the treatment of HER2-positive breast cancer.

In summary, strategic excision of the B and C rings, in concert with judicious linker engineering, markedly enhances C-terminal Hsp90 engagement, yielding analogues that combine sub-micromolar antiproliferative potency with substantially attenuated neurotoxicity. Carbon-mediated linkers—such as methyl-oxime and amide moieties—further potentiate HIF-1α suppression and improve tumor selectivity, whereas incorporation of heterocyclic nitrogen linkers enforces a “T-shaped” binding conformation that effectively destabilizes key client kinases without eliciting a compensatory heat-shock response.

### 4.2 Modification and antitumor activity of C′ derivatives of deguelin

**FIGURE 15:**
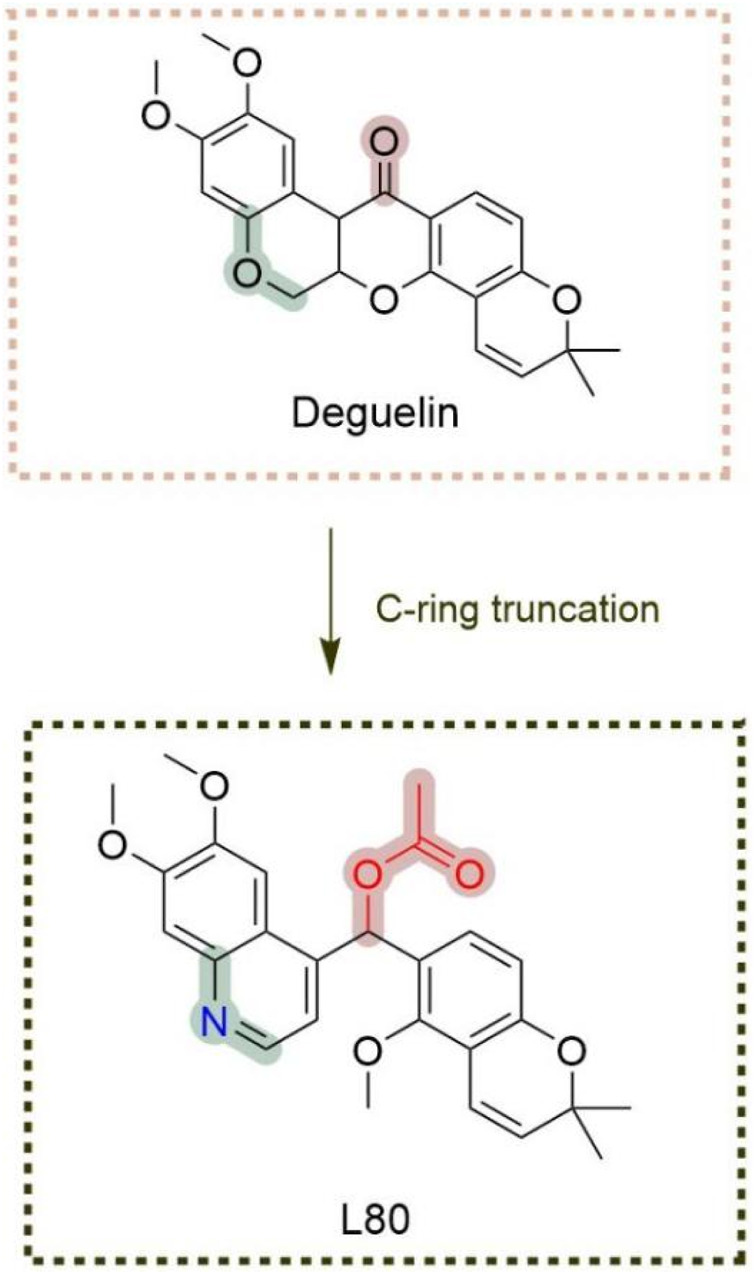
Novel deguelin C-ring-truncated derivative L80.

The C-ring truncated strategy was initially proposed and synthesized by Lee et al. (2015), who successfully developed and named a deguelin derivative, L80 (Figure 15), featuring a (6,7-dimethoxyquinolin-4-yl) (5-methoxy-2,2-dimethyl-2H-chromen-6-yl) methyl acetate moiety. Consistent with previous research on B- and C-ring truncations, the 2,2-dimethyl-2H chromene group of deguelin was preserved. The findings indicate that both deguelin and L80 can effectively penetrate the blood-brain barrier (BBB). At a maximum concentration of 10 μM, L80 exhibited no significant inhibitory effect on the viability of HT-22 cells. L80 demonstrated reduced toxicity to normal lung epithelial cells and retinal pigment epithelial cells. The antitumor effect of L80 in NSCLC cells was partially linked to enhanced apoptosis. L80 may regulate cellular processes related to the migration and invasion of NSCLC cells, including those exhibiting acquired resistance to paclitaxel. *In vitro* studies demonstrated that L80 significantly inhibited tumor growth in H1299 xenograft mice. Treatment with L80 resulted in a reduction of CD31 expression in tumors, potentially linked to the significant physical interaction between HIF-1α and Hsp90 observed in H1299 cells. The molecular docking results indicate that L80 binds competitively to the C-terminal ATP-binding site of Hsp90, stabilizing the open state of the Hsp90 homodimer through interactions with both the A and B chains. The findings indicate that L80 is a novel Hsp90 inhibitor characterized by low toxicity to normal cells and significant antitumor activity against NSCLC.

**FIGURE 16:**
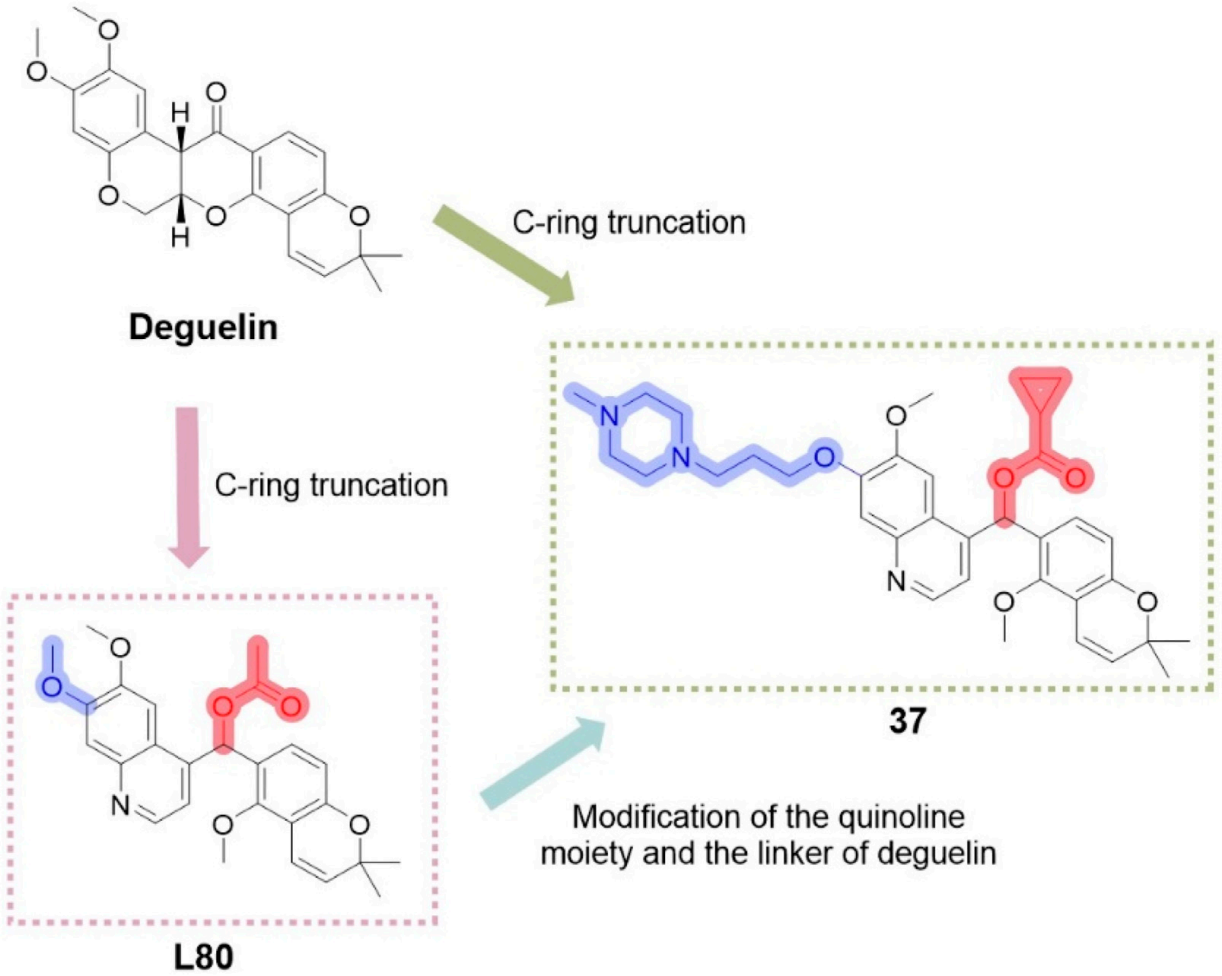
Design strategies for derivative **37** designed based on deguelin and L80.

This study has offered valuable insights for the design of subsequent compounds, thereby inspiring the development of more innovative derivatives. Nguyen et al. (2021) investigated modifications of the C-ring truncated scaffold of deguelin, emphasizing L80 and its derivatives. Their research preserved the quinoline core of L80 and examined a series of O-substituted analogs at the 7-O position of the quinoline ring, along with modifications to the acetoxymethylene linker of L80. The addition of diverse solubilizing side chains at the 7-O position significantly improved the pharmacokinetic and pharmacodynamic properties of the compounds. Compound **37** (Figure 16) demonstrated significant, concentration-dependent inhibition of cell viability in both trastuzumab-sensitive (BT474) and trastuzumab-resistant (JIMT-1) breast cancer cells, reducing cell viability to below 20% while exhibiting minimal cytotoxicity to normal cells. Compound **37** specifically targets the C-terminal binding site of HSP90, with its (S)-enantiomer demonstrating a superior binding profile to HSP90. Further evaluation suggests that the cytotoxicity of compound **37** in breast cancer cells is likely attributed to the destabilization and inactivation of HSP90 client proteins *via* its interaction with the C-terminal domain.

Overall, targeted removal of the C ring, complemented by installation of solubilizing side chains upon the quinoline nucleus, endows these derivatives with blood–brain barrier permeability while preserving robust C-terminal Hsp90 inhibition. Such modifications afford sub-micromolar cytotoxicity across both drug-sensitive and drug-resistant cancer cell models, yet incur negligible off-target neurotoxicity.

### 4.3 Modification and antitumor activity of B/C/E′ derivatives of deguelin

**FIGURE 17:**
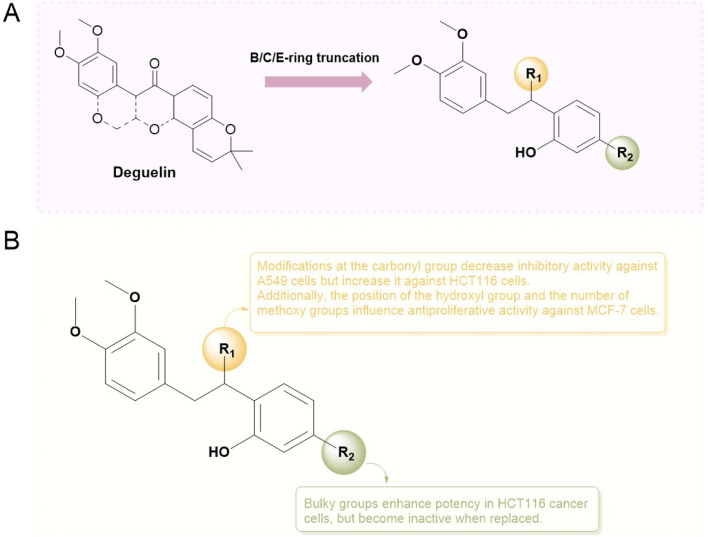
Modification areas of B/C/E-ring truncated derivatives of deguelin **(A)** and the SARs of it **(B)**.

John Alfon P. Francisco and colleagues (Francisco and Paderes, 2023) synthesized and evaluated fifteen deoxybenzoin derivatives through the truncation of the B/C/E rings. The ADME-Tox properties of these compounds were predicted using SwissADME18 and ProTox-II. The results indicated that none of the compounds violated Lipinski’s Rule of Five, exhibited good cell membrane permeability, and demonstrated favorable oral bioavailability, categorizing them as Class IV compounds (2,000 mg/kg > LD_50_ > 300 mg/kg). *In vitro* evaluations conducted on A549, HCT116, and MCF-7 cancer cell lines identified six compounds (**3a, 6a, 8c, 8d, 8e, and 8f**) exhibiting enhanced and selective antiproliferative activity. Compound 3a demonstrated specificity for A549 cells (IC_50_ = 6.62 μM), indicating a potency marginally greater than doxorubicin (IC_50_ = 7.38 μM) and similar to deguelin (IC_50_ = 6.47 μM). Conversely, the structural isomer, compound **5**, exhibited no inhibitory activity against A549 cells. Oxime derivatives showed limited effectiveness against A549 cells, yet were significant in inhibiting the proliferation of HCT116 cells. Compound **6a** demonstrated enhanced potency (IC_50_ = 3.43 μM) in comparison to deguelin and doxorubicin. Replacing the hydroxyl group in **6a** with methyloxime (IC_50_ = 93.3 μM) or benzyloxime (IC_50_ = 30.2 μM) resulted in a significant reduction in activity. Alkylation of the hydroxyl group on the D ring significantly improved the antiproliferative activity against HCT116 cells (IC_50_ = 68.7 μM). The potency of O-alkylated derivatives was enhanced by larger substituents, as demonstrated by compounds **8c** (IC_50_ = 13.3 μM) and **8d** (IC_50_ = 6.96 μM). The presence of hydroxyl and methoxy groups on the D ring increased the activity against MCF-7 breast cancer cells. As observed in HCT116 results, an increase in O-alkyl chain length enhanced efficacy, with compound **8c** (IC_50_ = 5.09 μM) demonstrating the greatest potency against MCF-7 cells, exceeding that of deguelin (IC_50_ = 33. μM) and doxorubicin (IC_50_ = 5.89 μM). In conclusion, the findings indicate that the synthesized deoxybenzoin derivatives possess considerable potential for further development as novel anticancer agents (Figure 17).

Collectively, complete ablation of the B, C and E′ rings from a deoxybenzoin backbone, combined with systematic variation in D-ring substituents and O-alkyl chain length, generates orally bioavailable analogues whose antiproliferative IC_50_ values rival or surpass those of parent deguelin. These findings underscore the critical role of tailored D-ring functionalization in driving anticancer efficacy.

### 4.4 Modification of other types of deguelin derivatives

**FIGURE 18:**
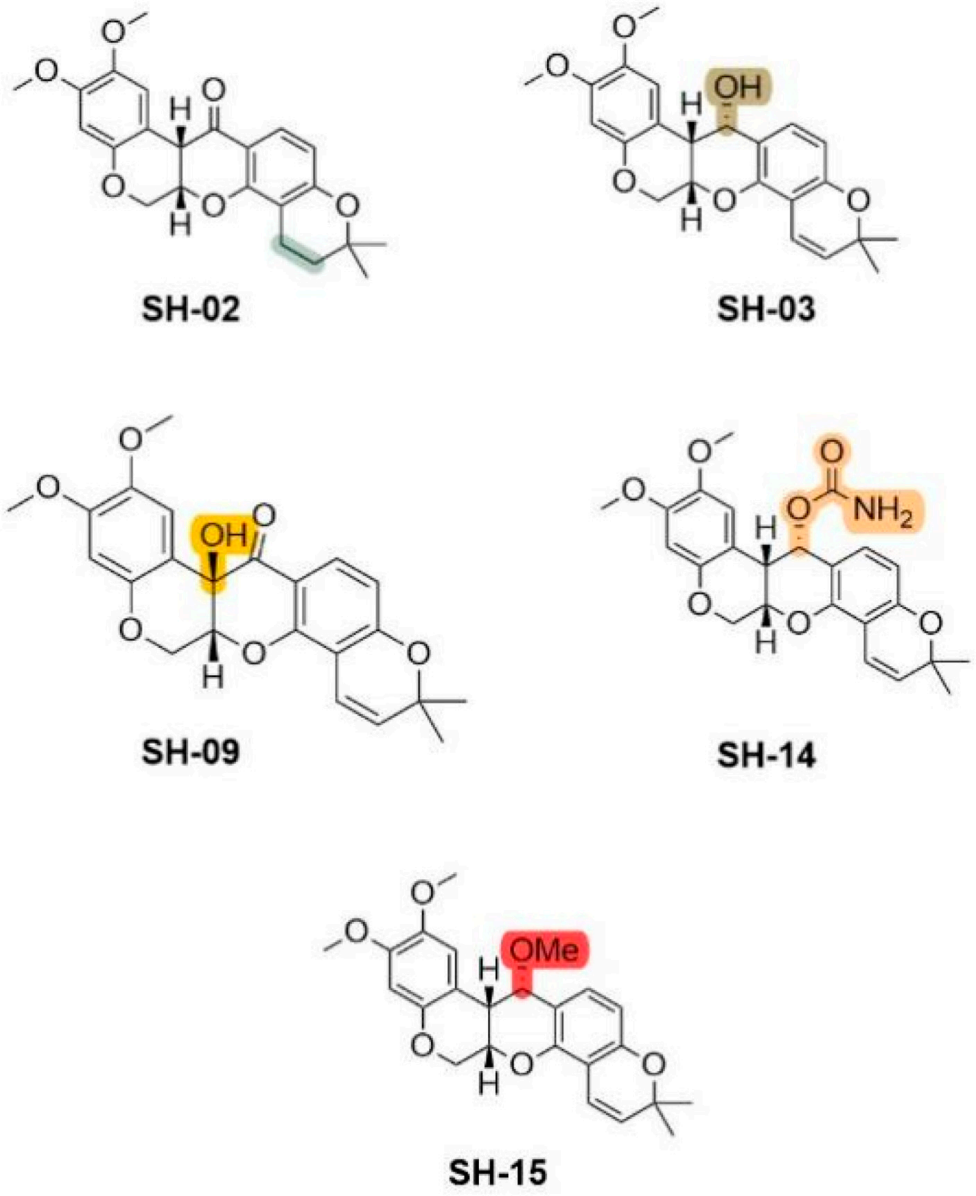
Unusual Deguelin derivatives obtained by retaining the intact skeleton.

The stability of deguelin is essential due to its susceptibility to decomposition when exposed to air and light (Cheng et al., 1972). The instability is attributed to its structural characteristics, specifically the acidic proton located at the tertiary carbon at the C7a position, which is in proximity to a ketone and a benzylic group. This configuration makes it vulnerable to nucleophilic attack induced by bases, resulting in the formation of an anion, or abstraction by radicals produced upon light exposure. Kim and colleagues (Kim et al., 2008) developed five deguelin derivatives by preserving the core skeleton, modifying the saturation of the E-ring, and adjusting the C7 position, such as by reducing the double bond in the E-ring. Under hypoxic conditions, the derivatives SH-01, SH-03, SH-09, SH-14, and SH-15 demonstrated effectiveness comparable to deguelin in inhibiting HIF-1α expression. Four derivatives, excluding SH-15, significantly inhibited the proliferation of H1299 and H460 NSCLC cell lines (Figure 18). Subsequent analysis revealed that treatment with deguelin, SH-02, SH-03, or SH-14 resulted in the downregulation of almost all proteins within the PI3K/mTOR signaling axis. The phosphorylated forms of 4E-binding protein and S6, important markers of translation signaling downstream of mTOR, displayed unique expression patterns following drug treatment. SH-14 demonstrated the most significant antitumor activity among the derivatives. In contrast to deguelin and other active derivatives, SH-14 did not significantly activate Src/STAT signaling. The carbamate moiety of SH-14 established additional hydrogen bonds with Lys112 and Asn1 residues on the binding pocket surface, leading to a binding energy score that is lower than that of deguelin or other derivatives. Solubility and cytotoxicity assessments indicated that SH-14 exhibited slightly lower lipophilicity (cLogP: 3.87) than deguelin (cLogP: 4.38) and may have reduced cytotoxicity towards normal lung epithelial cells.

The findings indicate that the novel derivative SH-14 demonstrates considerable potential as both a chemopreventive and therapeutic agent for cancer, exhibiting efficacy comparable to deguelin while presenting reduced toxicity. Further investigations are required to thoroughly assess its *in vivo* activity and neurotoxicity.

**FIGURE 19:**
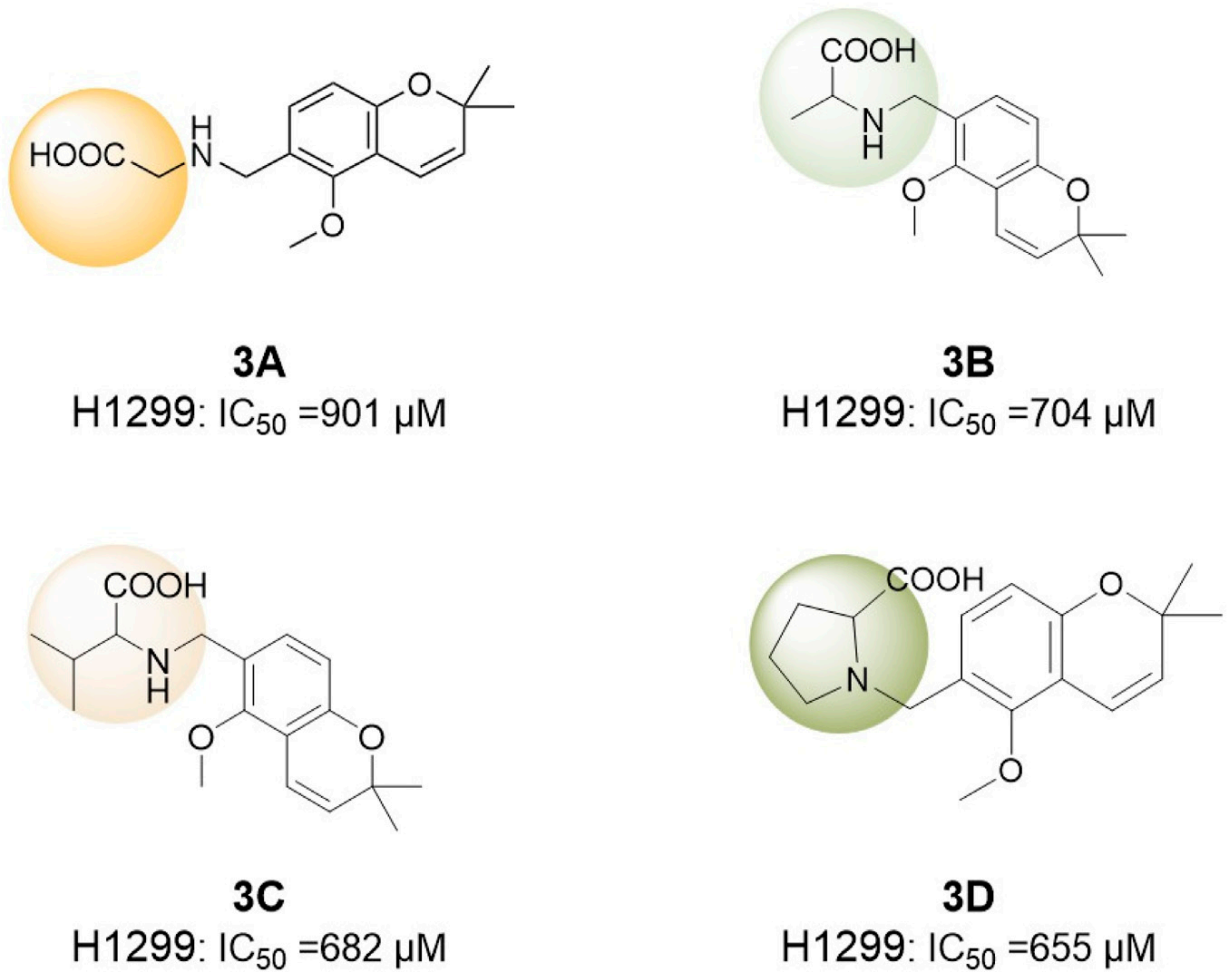
Truncated Deguelin derivatives modified by the Amino-acid-introduction strategy.

Conversely, Sun et al. (2017) investigated deguelin derivatives *via* ring-opening modifications. Their study was the first to integrate amino acids, recognized for their biocompatibility and cellular affinity, into the structure of deguelin. Due to the inherent rigidity of deguelin’s pentacyclic framework, which significantly resists conjugation with other bioactive moieties, the design focused solely on retaining the active region of deguelin. This fragment was subsequently conjugated with eight nonpolar amino acids, including glycine, L-alanine, and L-valine, to enhance membrane permeability and improve biological activity (Figure 19). The findings indicated that compounds **3C** and **3D** demonstrated enhanced inhibitory effects on H1299 cells relative to other derivatives. Among the compounds, **3C** exhibited greater pro-apoptotic activity compared to **3D**, while neither compound triggered apoptosis in normal human bronchial epithelial (HBE) cells. *In vitro* experiments demonstrated a concentration-dependent inhibition of zebrafish embryonic development by **3C** and **3D**, with **3C** exhibiting optimal inhibitory effects at 5 mM, resulting in a delay of embryo development to the shield stage. Furthermore, both compounds prompted apoptosis in zebrafish trunk and tail cells, with **3D** exhibiting a more significant effect. **3C** and **3D** demonstrated non-toxicity to normal HBE cells and SH-SY5Y neuronal cells, effectively addressing the neurotoxicity concerns linked to deguelin. The compounds exhibited toxicity to early-stage embryos; however, they were non-toxic to juvenile fish. The sectioning of brain tissue in juvenile fish demonstrated that **3C** and **3D** significantly reduced the neurotoxicity associated with deguelin.

Overall, Sun et al. effectively designed **3C** and **3D** derivatives by integrating amino acids into deguelin’s structure, resulting in improved membrane permeability and biological activity, alongside a reduction in neurotoxicity. This approach offers insights into the structural optimization of deguelin and establishes a foundation for developing novel low-toxicity deguelin-based therapeutics.

**FIGURE 20:**
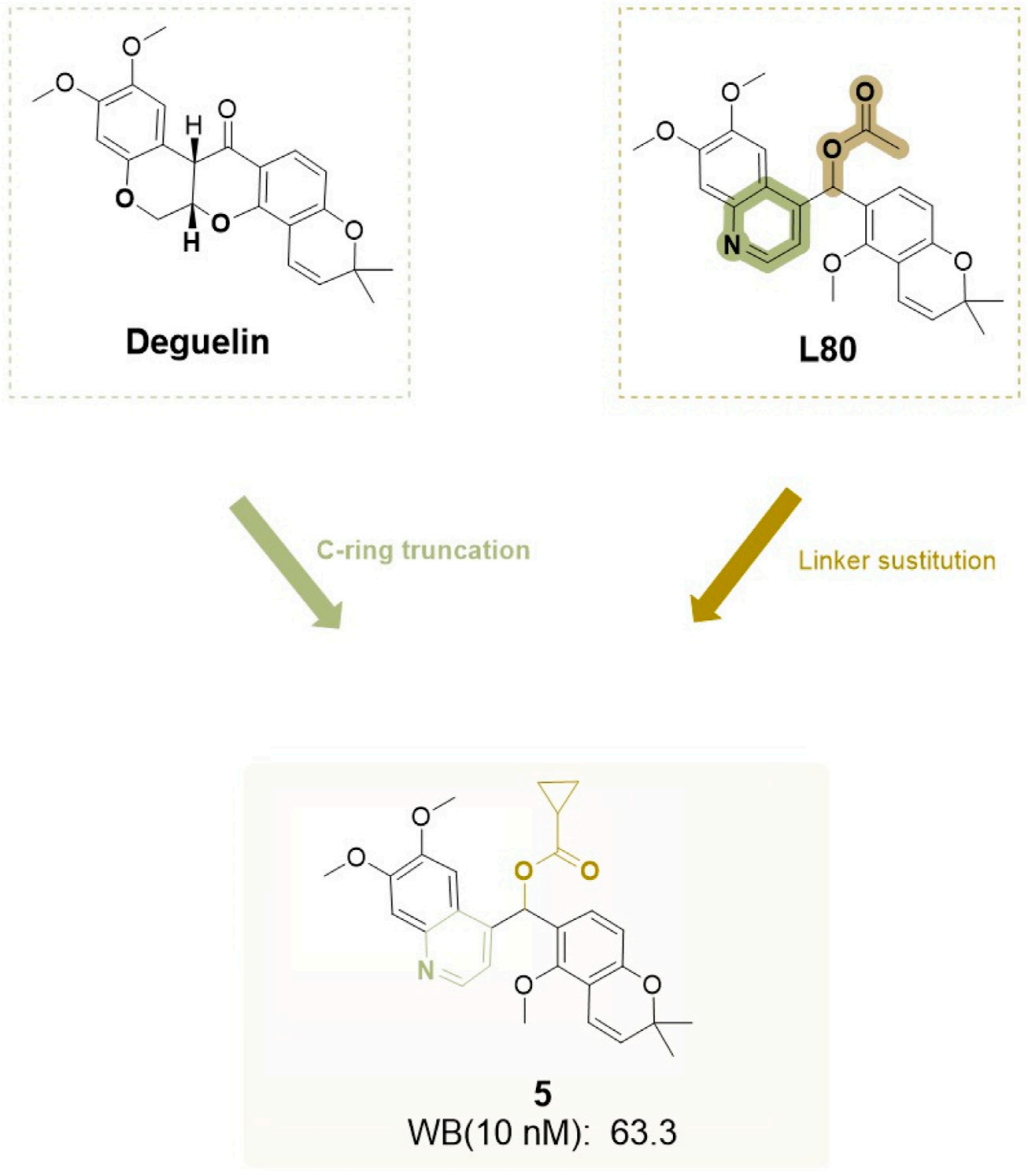
Design strategies for compound 5a designed based on L80.

**FIGURE 21:**
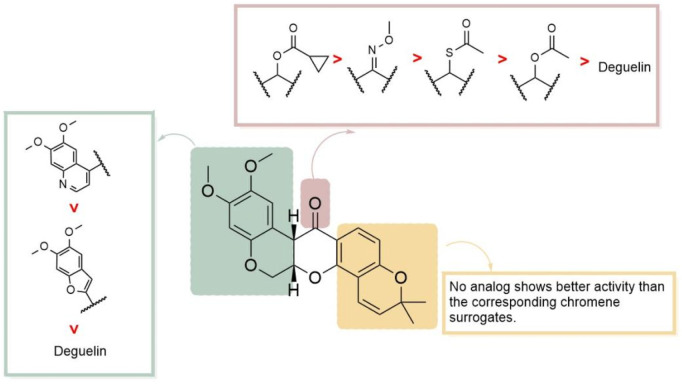
SAR of the novel HIF - 1α inhibitors derived from compound L80.

Based on L80, Kim and his team (Kim et al., 2016) designed a series of analogs by substituting the A/B-rings and D/E-rings with isosteric heterobicycles and modifying the linker with hydrogen-bonding groups. Initially, they fixed the A/B-ring as the 6,7-dimethoxyquinolin-4-yl moiety to investigate the effects of linker modifications. Among the derivatives, four compounds showed enhanced HIF-1α inhibition relative to deguelin: acetoxy (**2**, L-80) (cyclopropanecarbonyl)oxy (**5**) (Figure 20), acetylthio (**10**), and O-methyl oxime (**13**). Furthermore, two compounds exhibited activities similar to that of deguelin. Modifications to the D/E-ring proved less effective, as no compounds exceeded the activity of the corresponding chromene surrogates. The optimization of the A/B-ring revealed that 6,7-dimethoxyquinolin-4-yl and 5,6-dimethoxybenzofuran-2-yl analogs are promising structural motifs. At a concentration of 10 nM, four derivatives (**5**, **10**, **35**, and **37**) demonstrated superior inhibitory effects compared to deguelin, with compound 5 exhibiting the highest activity (WB, 10 nM: 63.3 nM). Cytotoxicity studies on NSCLC H1299 cells demonstrated that compound 5 produced dose-dependent antiproliferative effects. This compound also effectively inhibited retinal neovascularization in human retinal microvascular endothelial cells (HRMECs). The increased activity of compound 5 is attributed to its robust hydrogen bonding and hydrophobic interactions with the A/B-ring binding site. The methoxy group linked to the benzopyran ring establishes a hydrogen bond with Lys615 in chain A, whereas the cyclopropane ring occupies a hydrophobic pocket that interacts with Phe676 in chain A. The quinoline group establishes an additional hydrogen bond with the side-chain amide group of Arg612 in chain B and participates in an aryl-aryl hydrophobic interaction with Phe676 in chain B. Compound **5** interacts with the C-terminal ATP-binding pocket of the HSP90 homodimer in a manner akin to L-80, positioning it as a promising candidate for HIF-1α inhibition and subsequent development (Figure 21).

**FIGURE 22:**
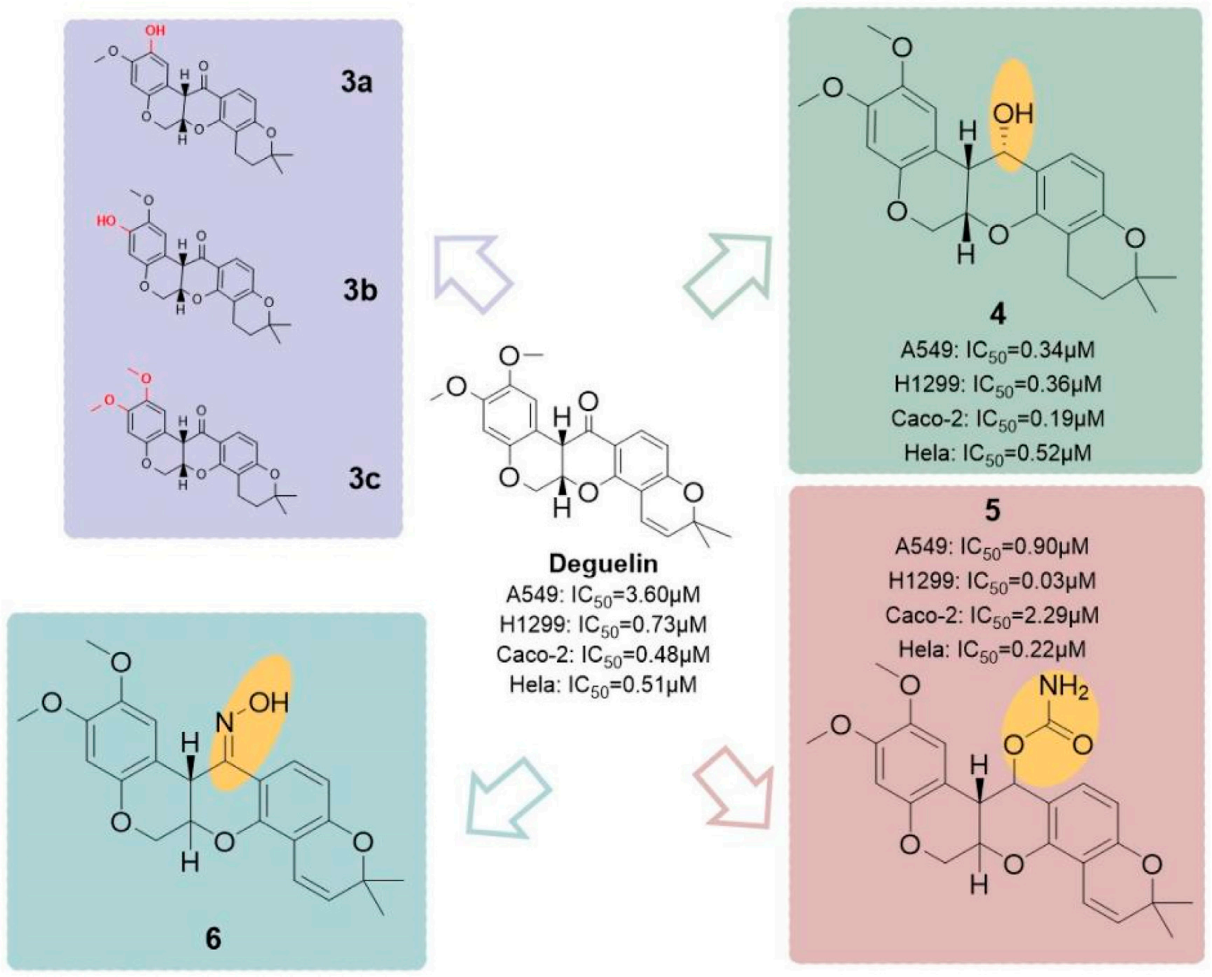
Deguelin derivatives with different substitutions on ring A and C7.

**FIGURE 23:**
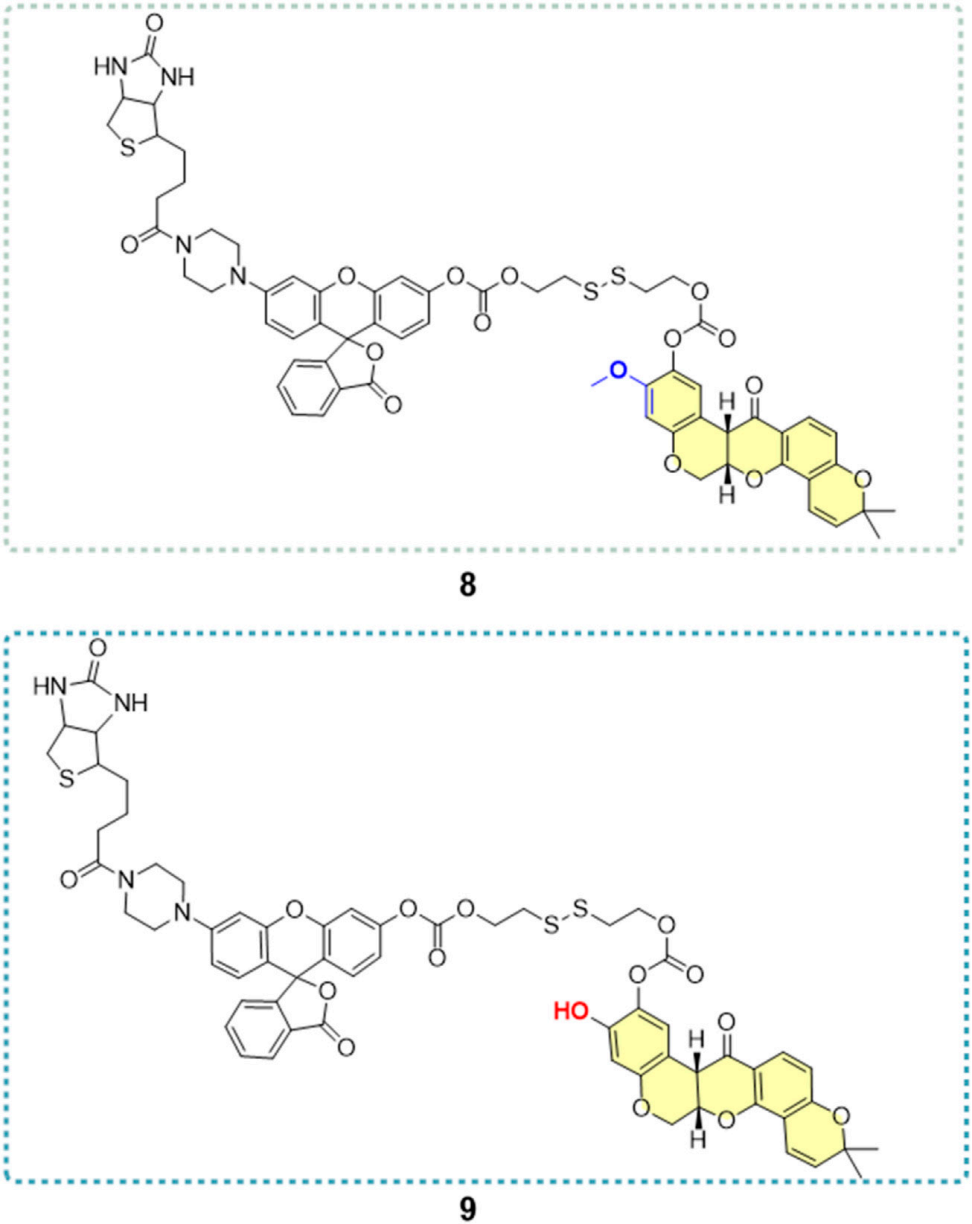
Novel deguelin derivatives 8 and 9 with targeted imaging function.

He and his colleagues (He, 2016) developed a series of derivatives by modifying the A-ring and the C7 position (Figure 22), resulting in six hydroxylated deguelin derivatives. Biological evaluations utilizing A549, H1299, Caco-2, and HeLa cell lines demonstrated that compounds **4** (IC_50_ = 0.36 μM) and **5** (IC_50_ = 0.03 μM) displayed enhanced inhibitory effects relative to deguelin (IC_50_ = 0.73 μM) across all four cancer cell lines, indicating that the C7 keto-hydroxyl moiety is essential to deguelin’s antitumor activity. In contrast, three A-ring methoxy-substituted derivatives (**3a, 3b, 3c**) exhibited enhanced inhibitory effects on H1299 cells, although their efficacy against the other 3 cell lines was inferior to that of the parent compound. This discrepancy suggests possible mechanistic differences in how these derivatives induce apoptosis in H1299 cells compared to other tumor cell types. The findings highlight the necessity for additional structural optimization at the C7 free hydroxyl group to improve therapeutic potential. The study also investigated the design and synthesis of novel targeted imaging derivatives by conjugating deguelin derivatives **3a** and **3c** with biotin, enabling the targeting of human NSCLC cells, along with fluorescent modules for imaging applications *via* cleavable linkers. Two derivatives, designated as **8** and **9** (Figure 23), were synthesized and assessed for their diagnostic and therapeutic efficacy against NSCLC. At concentrations of 10 μM and 1 μM, compounds **8** and **9** demonstrated significantly greater inhibitory activity against A549 cells compared to **3a** and **3c**. The results underscore the potential of these targeted imaging derivatives as novel theranostic agents, advancing the development of deguelin as a promising small-molecule therapeutic for cancer treatment.

## 5 Conclusion

Through comprehensive examinations of deguelin, substantial advancements have been made in elucidating its antitumor processes. Studies have shown that deguelin and its derivatives display significant antitumor efficacy in both *in vitro* and *in vivo* settings. Deguelin principally exerts its antitumor actions on malignant cells by inducing apoptosis, causing cell cycle arrest, blocking angiogenesis, slowing cell migration, and regulating metabolic pathways. These effects are essential in suppressing tumor cell proliferation and viability. They also aid in the prevention of tumor metastasis. Deguelin, as a natural anti-cancer chemical, offers prospective benefits for targeting diverse cancer cell types. It impedes cancer cell proliferation by modulating several critical oncogenic pathways (Wang et al., 2013), including the PI3K-Akt, HIF-1-VEGF, IKK-IKBa-NF-κB, EMT, and AMPK-mTOR-survivin signaling cascades (Yang et al., 2013). These findings establish a robust scientific basis for the advancement of deguelin and its derivatives as prospective chemopreventive and chemotherapeutic medicines.

Moreover, our comprehension of the structural optimization of deguelin and the SAR of its derivatives has significantly advanced. The research conducted by Jee Woo Lee and Ho-Young Lee’s team has significantly advanced the study of deguelin among various investigations. In conclusion, the inflexible pentacyclic configuration of deguelin presents considerable obstacles for conjugation with other bioactive compounds, making ring-opening alterations a viable strategy to augment its biological efficacy. Nevertheless, the DE-ring framework is essential for preserving the compound’s overall effectiveness. The methoxy groups on the A-ring are essential for action, and the linking chain between the B and C rings is significant, but some design flexibility is permitted. Alterations at the C7 position are crucial, as changes at this locus markedly influence the compound’s efficacy in inhibiting cancer cell proliferation. The physicochemical and biological characteristics of the derivatives are significantly affected by the kind and location of the attached functional groups.

Despite comprehensive study on structural-activity correlations and cytotoxic consequences, a significant element that has been neglected is the possible neurotoxicity linked to the usage of deguelin and its derivatives. Despite extensive study on the cytotoxic effects of deguelin and its derivatives, there is a significant deficiency in studies investigating its potential neurotoxicity, underscoring a crucial gap of the existing literature. In conclusion, the structural modification of deguelin presents several opportunities to augment its synthetic accessibility, diminish toxicity, and boost its bioactivity. Beyond these, other critical bottlenecks include often limited systemic bioavailability and suboptimal pharmacokinetic profiles for many natural products, the potential for off-target toxicities aside from neurotoxicity, and the manufacturing scalability of structurally complex derivatives. The successful clinical translation is also hindered by the common translational gap between preclinical models and human efficacy, and a current lack of predictive biomarkers for patient stratification.

Therefore, future investigations should strategically address these limitations. To mitigate toxicity while preserving antitumor efficacy, continued efforts in structural modification are paramount, focusing on enhancing selectivity, particularly for the Hsp90 C-terminal domain to avoid the heat shock response, and reducing off-target interactions. The development of simplified analogs with improved synthetic accessibility and potentially better pharmacological properties should be pursued. Combination therapies with other established anticancer agents may also allow for dose reduction and synergistic effects, thereby improving the therapeutic index. To address instability and improve bioavailability, innovative drug delivery systems, such as nanoparticle carriers, liposomal formulations, polymer conjugates, or prodrug strategies, warrant further exploration. A thorough preclinical safety and toxicology profiling, including long-term studies, for the most promising derivatives is crucial, especially to address the current gap in understanding the neurotoxicity of these modified compounds. Concurrently, the discovery and validation of biomarkers will be essential for identifying patient populations most likely to benefit from deguelin-based therapies in future clinical trials.

In conclusion, deguelin, a highly potent antitumor compound belonging to the rotenone class of flavonoids, harbors great potential in research and clinical applications. As continuous progress is made in elucidating its action mechanisms and innovating structural modifications, there is a strong likelihood that deguelin will become a key therapeutic agent in the treatment of cancer and other diseases in the future.

## Glossary

HSPs: Heat-shock proteins
Hsp90: heat shock protein 90
PD: Parkinson’s disease
SAR: structure-activity relationship
NSCLC: non-small cell lung cancer
LCCs: lung cancer cells
GBM: Glioblastoma multiforme
MMPs: matrix metalloproteinases
HUVECs: Human umbilical vascular endothelial cells
PDXs: patient-derived xenografts
EGFR: epidermal growth factor receptor
VEGF: vascular endothelial growth factor
VEGFR: vascular endothelial growth factor receptor
AMPK: AMP-activated protein kinase
PI3K: phosphatidylinositol 3-kinase
FAK: focal adhesion kinase
mTOR: mammalian target of rapamycin
MAPK: mitogen-activated protein kinase
NF-κB: nuclear factor-κB
ERK: extracellular signal-regulated kinase
HIF-α1α: hypoxia-inducible factor-1α
FAK: focal adhesion kinase
PECAM-1: platelet endothelial cell adhesion molecule-1
Nek2: NIMA-related kinase 2
PUMA: p53 upregulated modulator of apoptosis
Bax: bcl- 2-associated x
klf: Kruppel like factor
EMT: pithelial mesenchymal transition
JAK2: Janus kinase 2
Rho A: Ras homology family member A
ROS: reactive oxygen species
HR: homologous recombination
ICAM: intracellular adhesion molecule 1
NTD: N-terminal ATP-binding domain
MD: the middle co-chaperone and client-binding domain
CTD: C-terminal dimerization domain
BBB: blood-brain barrier
